# The CHROMEVALOA Database: A Resource for the Evaluation of Okadaic Acid Contamination in the Marine Environment Based on the Chromatin-Associated Transcriptome of the Mussel *Mytilus galloprovincialis*

**DOI:** 10.3390/md11030830

**Published:** 2013-03-12

**Authors:** Victoria Suárez-Ulloa, Juan Fernández-Tajes, Vanessa Aguiar-Pulido, Ciro Rivera-Casas, Rodrigo González-Romero, Juan Ausio, Josefina Méndez, Julián Dorado, José M. Eirín-López

**Affiliations:** 1 Chromatin Structure and Evolution Group (CHROMEVOL-XENOMAR), Department of Cellular and Molecular Biology, University of A Coruna, E15071 A Coruna, Spain; E-Mails: v.ulloa@udc.es (V.S.-U.); jfernandezt@udc.es (J.F.-T.); crivera@udc.es (C.R.-C.); rgonzalezr@udc.es (R.G.-R.); fina@udc.es (J.M.); 2 Department of Biological Sciences, Florida International University, North Miami, FL 33181, USA; 3 Wellcome Trust Center for Human Genetics, University of Oxford, OX3 7BN Oxford, UK; 4 Artificial Neural Networks and Adaptive Systems Laboratory (RNASA-IMEDIR), Department of Information and Communication Technologies, University of A Coruna, E15071 A Coruna, Spain; E-Mails: vaguiar@udc.es (V.A.-P.); julian@udc.es (J.D.); 5 Department of Biochemistry and Microbiology, University of Victoria, V8W 3P6 Victoria BC, Canada; E-Mail: jausio@uvic.ca

**Keywords:** okadaic acid, Harmful Algae Blooms, mussels, chromatin, database, *Mytilus galloprovincialis*

## Abstract

Okadaic Acid (OA) constitutes the main active principle in Diarrhetic Shellfish Poisoning (DSP) toxins produced during Harmful Algal Blooms (HABs), representing a serious threat for human consumers of edible shellfish. Furthermore, OA conveys critical deleterious effects for marine organisms due to its genotoxic potential. Many efforts have been dedicated to OA biomonitoring during the last three decades. However, it is only now with the current availability of detailed molecular information on DNA organization and the mechanisms involved in the maintenance of genome integrity, that a new arena starts opening up for the study of OA contamination. In the present work we address the links between OA genotoxicity and chromatin by combining Next Generation Sequencing (NGS) technologies and bioinformatics. To this end, we introduce CHROMEVALOAdb, a public database containing the chromatin-associated transcriptome of the mussel *Mytilus galloprovincialis* (a sentinel model organism) in response to OA exposure. This resource constitutes a leap forward for the development of chromatin-based biomarkers, paving the road towards the generation of powerful and sensitive tests for the detection and evaluation of the genotoxic effects of OA in coastal areas.

## 1. Introduction

Massive algal proliferations are among the most important sources of contamination in the sea. These episodes may arise as a consequence of either natural or anthropogenic causes, leading to large accumulations of algae in the marine environment [1]. Quite often, massive algal proliferations include blooms of toxin-producing organisms known as Harmful Algal Blooms (HABs), producing high concentrations of potentially harmful biotoxins that are accumulated throughout the food chain. Among HAB biotoxins, Diarrhetic Shellfish Poisoning (DSP) toxins are especially predominant across European coasts, causing alterations in the gastrointestinal system of human consumers of contaminated shellfish [2,3]. The main active principle in DSPs is Okadaic Acid (OA) [4], which is synthesized by dinoflagellates of the genera *Dinophysis* and *Prorocentrum* [5]. OA has genotoxic potential, constituting a tumor promoter and apoptosis inducer able to cause DNA oxidative damage [6,7]. Particularly, DNA Double Strand Breaks (DSBs) stand out for their severity among the genotoxic effects exerted by OA and require the activation of prompt repair mechanisms in order to avoid serious damage in the cell [8,9].

During the last 30 years, fisheries and aquaculture-based industries have experienced important economic losses due to the dramatic increase in the diversity of toxic algal species and the toxins they produce [10], constituting a serious threat for human consumers [1]. Consequently, a very important effort has been devoted to OA biomonitoring in estuarine areas by using sentinel organisms, most notably bivalve molluscs [9,11]. These studies have progressively transitioned from traditional biomonitoring methods (based on physicochemical and physiological parameters) to more sensitive molecular probes [12,13,14,15]. Given the role of chromosomal proteins in the modulation of chromatin structure and DNA metabolism (including DNA repair) [16], the study of chromatin-associated biomarkers constitutes a powerful and sensitive approach for the evaluation of genotoxicity. The usefulness of chromatin-based genotoxicity tests has already been demonstrated in mammals, where histone H2A.X phosphorylation has been used to assess the extent of DNA repair following exposure of cells to DNA-damaging agents [17,18,19]. Yet, this approach is largely unexplored in those organisms where chromatin information is scarce, including bivalve molluscs [20]. Furthermore, the lack of knowledge regarding gene and protein sequences in these organisms constitutes a very important barrier for the analysis of high-throughput -omic data, especially as it pertains to data assembly and annotation of highly divergent and/or lineage-specialized genes [20,21,22,23]. Even though the genome sequence of the Pacific oyster *Crassostrea gigas* has been recently published [24], the amount of information available for marine bivalves remains scarce compared to other model organisms in spite of their environmental value. 

In the present work we specifically address the links between OA genotoxicity and potential chromatin-associated biomarkers by combining Next Generation Sequencing (NGS) technologies and bioinformatics. To this end, we introduce CHROMEVALOAdb [25], a database containing the chromatin-associated transcriptome of the mussel *Mytilus galloprovincialis* in response to OA exposure. The information provided in this database includes fully traceable raw ESTs assembled into consensus sequences and classified into unigenes linked to Gene Ontology (GO) information (function, process and subcellular compartment) as well as to expression information in response to OA. CHROMEVALOAdb allows for the manual browsing and keyword-based search of chromatin-associated contigs. In addition, the whole OA-specific transcriptome can be accessed by using built in BLAST and CLUSTAL W tools. Overall, the present work constitutes a leap forward in the study of the genotoxic effect exerted by OA in these organisms, paving the road towards the development of chromatin-based tests for detecting and evaluating the genotoxic effect of OA in the marine environment.

## 2. Results and Discussion

### 2.1. Sequencing and Annotation of OA-Specific ESTs in *M. galloprovincialis*

**Figure 1:**
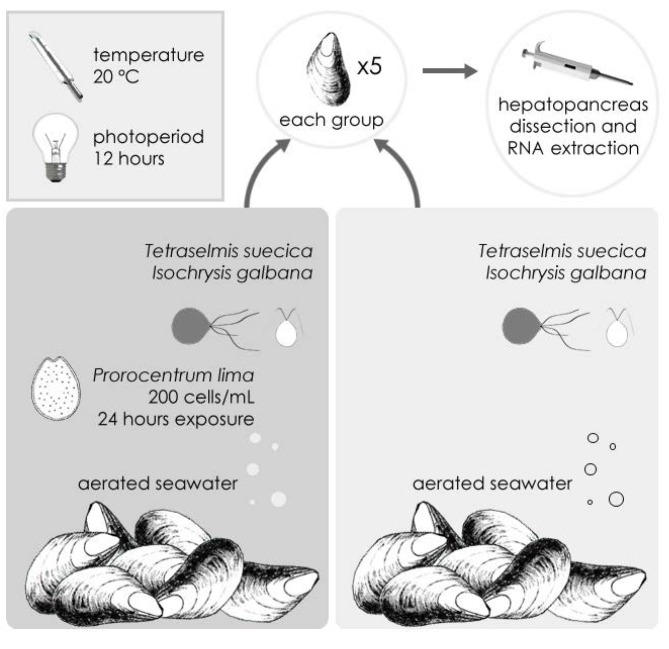
Experimental settings for the exposure of mussels to Okadaic Acid (OA), specifying the environmental conditions for treated (additionally fed with OA-producing microalgae *P. lima*) and control groups of mussel individuals.

Mussels (*M. galloprovincialis*) were sampled in an area of the Galician coast (northwest Spain) subject to a low impact of dinoflagellate blooms. Specimens were experimentally exposed to OA in the laboratory (Figure 1, see Experimental Section) using a set of conditions that were previously proven to cause significant genotoxic damage (200 cells/mL of the OA-producing dinoflagellate *Prorocentrum lima*, 1 day exposure) [9,26]. The accumulation of OA in digestive gland tissue was subsequently confirmed by HPLC-MS quantification (Table 1). 

**Table 1:** HPLC-MS quantification of OA in digestive gland tissue.

Experimental conditions	OA-content (ng/g)
Control	Below detection limit (~0)
OA-exposed	18.27

Raw normalized libraries constructed from mussel specimens exposed and non-exposed to OA were sequenced using pyrosequencing technology at 40× depth, producing 493,440 and 491,109 raw reads for the control (NORM_MGC) and the OA-exposed (NORM_MGT) libraries, respectively. These data allowed the assembly of 16,395 consensus sequences in the case of the control library and 24,624 consensus sequences from the OA-exposed library, with average length values of 712 and 644 bp, respectively. Approximately 44% of the assembled sequences (17,952) were annotated by using BLAST (blastx) homology searches against non-redundant (nr) protein databases, including 7335 contigs in the control library and 10,617 contigs in the OA-exposed library (38% and 45%, respectively), setting an expectation (*e*) value of 1 × 10^−6^ or better (Table 2).

**Table 2:** Amount of data in each step of the data processing pipeline.

Library	Reads	Contigs	Annotated Sequences
NORM_MGC (control)	493,440	16,395	7335
NORM_MGT (OA-exposed)	401,109	24,624	10,617
	**Contigs**	**Unigenes**	**Differentially Expressed**
TOTAL	41,019	2131	1254
CHROMATIN-ASSOCIATED	14,480	1124	90

### 2.2. Novel Chromatin-Associated Transcripts in CHROMEVALOAdb

Chromatin-associated transcripts were identified from the assembled OA-specific transcriptome from *M. galloprovincialis* by following two complementary strategies (see Experimental Section for details). On one hand, a list of keywords identifying chromatin-associated components was used to screen annotated transcripts regarding sequence description and related gene ontology terms (Supplementary Figures S1 and S2). On the other hand, BLAST homology comparisons were performed against specialized chromatin databases. The combination of both strategies resulted in the identification of 14,480 chromatin-associated contigs in control and OA-exposed libraries among which 1124 were identified as chromatin-associated unigenes (Table 2). The analysis of gene expression profiles (Supplementary Figure S3) allowed us to define groups of statistically significant unigenes upregulated and downregulated in the presence of OA (a total number of 1254) among which 90 were identified as chromatin-associated (Table 2). This information, along with gene ontology and expression profile data, constitutes the core of CHROMEVALOAdb.

**Figure 2:**
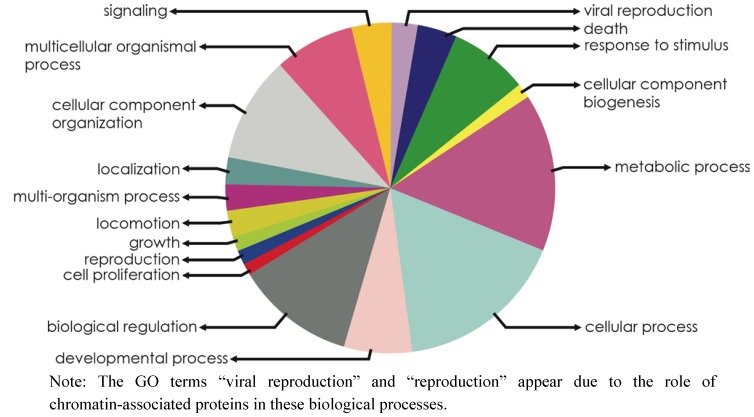
Biological processes on which chromatin-associated unigenes could be potentially involved during the response to OA.

The ontological analysis of the biological processes on which the identified chromatin-associated unigenes could be potentially involved revealed that cellular and metabolic processes are most significantly deregulated in response to OA (Figure 2). Furthermore, a significant deregulation of genes involved in chromatin remodeling (inhibited) and transmembrane transport (overexpressed) was identified through global ontological analyses based on the whole OA-specific transcriptome (Fisher’s exact test approach using topGO R-bioconductor package, Supplementary Figure S4). Even though additional experimental studies will be needed to decipher the functional role of chromatin-associated unigenes in response to OA, these results may be indicative of an activation in protective detoxifying mechanisms in mussels after one day of exposure to OA, once DNA has been repaired.

Comparisons between OA-specific EST information from CHROMEVALOAdb and *Mytilus* ESTs information from the MytiBase EST knowledge database [27] revealed that approximately 25% of the chromatin-associated sequences contained in CHROMEVALOAdb are redundant with MytiBase sequences. This extends also to the case of the complete OA-specific transcriptome, with a 30% of the ESTs being redundant with MytiBase sequences considering no identity cutoff value (manuscript in preparation). In other words, approximately 75% of the ESTs contained in CHROMEVALOAdb constitute previously unknown transcripts in the mussel *M. galloprovincialis*, establishing a very important contribution not only for the study of OA chromatin-associated biomarkers, but also for the characterization of the mussel genome.

### 2.3. Availability, Management and Application of Data Stored in CHROMEVALOAdb

**Figure 3:**
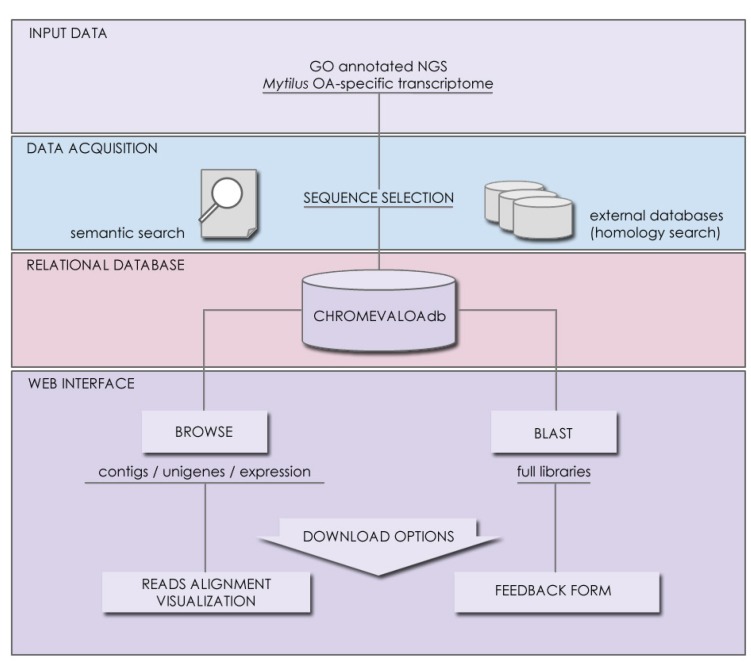
Diagram showing the pipeline of data management in CHROMEVALOAdb. Starting from files containing the fully annotated transcript libraries, the selection of chromatin-associated sequences is carried out through semantic and homology search approaches. Sequences and annotations are organized in the relational structures of the database and made available through web interface, including data retrieval and feedback utilities.

Management of data quality constitutes a basic requirement of NGS projects that is often overlooked, resulting in the loss of important information for fine sequence curation and identification of DNA polymorphisms, among other quantitative analyses. The structure of CHROMEVALOAdb strengthens this aspect by providing full access to raw reads used to assemble the consensus sequences annotated in the database. This feature facilitates the alignment of quality-filtered raw sequences, establishing links with specific expression patterns in response to OA. Furthermore, the availability of the full dataset of contigs allows users to retrieve anonymous sequences by using the BLAST tool interface and communicate new chromatin-associated findings through a standardized feedback form, contributing to the curation of the information in CHROMEVALOAdb. Processed data, on the other hand, is also downloadable as flat text files containing information that can be filtered by keywords (Figure 3).

**Figure 4:**
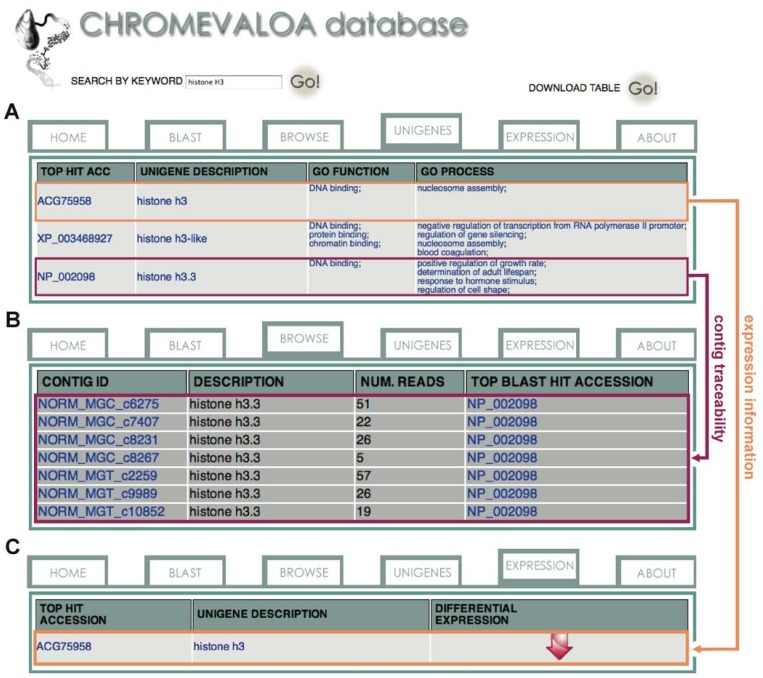
Chromatin-associated sequence query and results. CHROMEVALOAdb provides access to a search engine allowing users to find transcripts differentially expressed in response to OA. (**A**) Searches can be performed on the basis of sequence homology (BLAST) or keywords. (**B**) Results from individual unigenes provide gene ontology information as well as details on the contigs included in a given unigene. (**C**) Differential expression information (upregulated and downregulated transcripts) for the chromatin-associated unigenes is presented through an intuitive format using arrow icons.

The information contained in CHROMEVALOAdb serves a dual purpose. First, it helps identify previously unknown chromatin-associated transcripts in the mussel *M. galloprovincialis*, specially histone variants and chromatin remodeling factors (Figure 4A,B). This aim is motivated by the role of chromatin-associated proteins in the maintenance of genome integrity, most notably in the case of DNA DSB repair [20,23]. Within this context, the generation of new molecular data and its organization in CHROMEVALOAdb helps increase the knowledge about mollusc chromatin, setting up a framework for studying its role in DNA repair. The second purpose of CHROMEVALOAdb is to establish cause-effect relationships between OA exposure and specific expression patterns of chromatin-associated factors involved in the maintenance of genome integrity. This approach will help identify potentially sensitive biomarkers of OA genotoxic effect. To this end, CHROMEVALOAdb provides differential expression information for chromatin-associated unigenes, using an intuitive graphical format based on arrows (up-regulated and down-regulated transcripts, Figure 4C). The combination of the newly characterized DNA sequences together with their associated expression information in response to OA paves the road towards the development of chromatin-based tests for detecting and evaluating the genotoxic effect of OA in the marine environment.

## 3. Experimental Section

### 3.1. Synthesis of ESTs Libraries and Transcriptome Assembly

Mussel specimens (*M. galloprovincialis*) were sampled in Valcobo beach, Galicia (northwest coast of Spain, 43°19′02.71″N 8°21′56.35″W) and immediately transported to the laboratory thereafter where they were maintained under controlled light/temperature conditions and fed with a standard mixture of the microalgae *Isochrysis galbana* and *Tetraselmis suecica* (Figure 1). Individuals were subsequently divided into a control group and a group exposed to OA that was additionally fed with a culture of the DSP-producing microalgae *P. lima* (200 cells/mL for 24 h). The quantification of OA in digestive gland tissue was performed by using high performance liquid chromatography coupled to mass spectrometry (HPLC-MS). Extraction of mRNA was subsequently performed from pooled digestive gland tissue (hepatopancreas) from five individuals in each group. The choice of this tissue as mRNA source is motivated by its ability to accumulate the biotoxin in large amounts and its detoxifying role in mussel metabolism [28]. 

cDNA libraries were synthesized using the SMARTerTM PCR cDNA synthesis kit (Clontech, Mountain View, CA, USA) with an extra purification step using GeneJET™ PCR Purification Kit (Thermo Scientific, Waltham, MA, USA), and normalization was performed following the protocol of the Trimer cDNA Normalization Kit (Evrogen, Moscow, Russia). Libraries were sequenced using Roche-454 FLX+ Titanium pyrosequencing, obtaining both exposed and control datasets. Reads from both libraries were pre-processed (quality filtering and contaminantion removal) by combining the CD-HIT-454 [29] and the BLAST+ software [30] implemented in the SeqtrimNext pipeline [31], as well as the Cutadapt v1.0 software [32]. Sequence assembly was carried out using MIRA v.3.4.0 sequence assembler [33]. The sequences described in this work are available at the Sequence Read Archive (SRA) database under the accession number SRA056210.

### 3.2. Database Contents, Accessibility and Tool Implementation

The relational structure of CHROMEVALOAdb was developed using MySQL, allowing full traceability of raw ESTs from consensus sequences of individual genes. Contigs are classified into unigenes to eliminate redundancy based on BLAST analysis parameters (same top blastx hit, mean similarity larger than 80% and an *e*-value below 1 × 10^−10^). The descriptions of the unigenes are linked to their corresponding contigs and to ontology annotations. All the information stored in CHROMEVALOAdb is freely available for browsing and downloading without login or registering requirements. The information gathered by CHROMEVALOAdb is managed through Perl-written Common Gateway Interfaces (CGIs) that communicate with the Relational Database Management System (RDBMS) MySQL using Perl’s database interface (DBI) module. Server-side tools for sequence alignment, data visualization and result formatting/retrieval are administered by built in HTML web interfaces. BLAST results are formatted and interactively presented in HTML format including graphics, using Bioperl packages. Multiple sequence alignments are generated using CLUSTAL W [34] and displayed with an embedded applet of the alignment editor Jalview [35,36]. Local data is linked to reference public databases such as NCBI repositories for extended homolog sequence descriptions and AmiGO [37] for gene ontology term definitions. 

### 3.3. Gene Annotation and Expression Analysis

The functional annotation of the consensus read assemblies was carried out using the Blast2GO suite [38], combining Gene Ontology (GO), InterProScan (IPS) protein domain information [39] and annotation enrichment using ANNEX [40]. Additionally, full-length transcripts were subsequently identified using the Full-Lengther tool [41]. Identification of chromatin-associated transcripts was subsequently implemented following two complementary strategies. First, a keyword-based routine was defined to identify chromatin-associated transcripts among sequence descriptions and related ontology terms (Supplementary Materials S1 and S2). Secondly, BLAST (blastn and blastx) homology searches were performed against the Histone Database [42], as well as against ChromDB [43] and CREMOFAC [44] databases, setting an *e*-value threshold of 1 × 10^−10^. Functionally annotated and classified sequences, along with relevant metadata, are organized and stored in CHROMEVALOAdb.

The biological processes on which the identified chromatin-associated unigenes could be potentially involved were studied by performing ontological analyses based on GO terms (Supplementary Figure S3). Expression profiles in response to OA were further studied by comparing control and OA-exposed libraries, using the edgeR package from R-Bioconductor [45] with the False Discovery Rate (FDR) threshold set to 0.1 (Supplementary Figure S4). Read count for each assembled sequence was performed using SQL-based queries on the raw data contained in CHROMEVALOAdb. This approach allowed us to define groups of statistically significant unigenes upregulated and downregulated in the presence of OA. 

## 4. Conclusions

CHROMEVALOAdb provides a powerful resource to investigate the molecular basis underlying the genotoxic effect of OA in mussels and for understanding the chromatin-associated mechanisms that counteract the harmful effect of this toxin in these organisms (*i.e.*, mechanisms involved in DNA repair). Furthermore, it allows the establishment of cause-effect relationships between OA and the differential expression of chromatin-associated factors involved in DNA DSB repair, helping to identify potential sensitive biomarkers for the development of chromatin-based OA genotoxicity tests. The implementation of these tests in natural populations has critical implications for the evaluation of DNA damage in commercially relevant organisms, the optimization of their harvesting and the elaboration of additional tests designed to evaluate the safety of their consumption and potential implications for consumer’s health. The design of CHROMEVALOAdb sets the basis for the future integration of model-based and semi-automated curation systems. In addition, the characterization of additional transcriptomes (*i.e.*, at different stages of the genotoxic stress and in different tissues), together with data integration and workflow automation for interactome network development, constitute future objectives for the improvement of the database. Altogether, these approaches will help increase the knowledge of the chromatin-associated mechanisms involved in the response to the genotoxic effect of OA, by using Knowledge Discovery in Databases (KDD) techniques.

## Supplementary Files

Supplementary File 1 Supplementary Materials (PDF, 526 KB)
