# Supplementary material for: The CHROMEVALOA Database: A Resource for the Evaluation of Okadaic Acid Contamination in the Marine Environment Based on the Chromatin-Associated Transcriptome of the Mussel Mytilus galloprovincialis
Source: Mar Drugs. 2013 Mar 12;11(3):830–41. doi: 10.3390/md11030830 (PMC3705373; doi:10.3390/md11030830)
Supplement: Supplementary File 1 — Supplementary Materials (PDF, 526 KB) [file marinedrugs-11-00830-s001.pdf]

Supplementary Materials

Figure S1. List of keywords used to identify chromatin-associated transcripts in the assembled OA-specific transcriptome from *M. galloprovincialis*. **2**

Figure S2. Script used to implement a keyword-based routine for the identification of chromatin-associated transcripts among sequence descriptions and related ontology terms. **3**

Figure S3. Differential expression analysis results displaying unigenes with False Discovery Rate (FDR) < 0.1. **4**

Figure S4. Gene Ontology (GO) terms with highest significance levels (Fisher's exact test) in (**A**) upregulated and (**B**) downregulated unigenes. **33**

Figure S1. List of keywords used to identify chromatin-associated transcripts in the assembled OA-specific transcriptome from *M. galloprovincialis*.

Acf
Centromere
Chd
Chrac
Chromatin
Dna binding
Dna damage
Dna repair
Double strand break
Epigenetic
High mobility group
Histone
Hmg-a,b,n
Ino80
Mi-2
Nucleosome
Nurf
Post translational modification
Protamine
Rsc
Rsf
Saga
Swi/snf

Figure S2. Script used to implement a keyword-based routine for the identification of chromatin-associated transcripts among sequence descriptions and related ontology terms.

```
#! /usr/bin/perl -w

#Victoria Suárez-Ulloa
#Selection of annotated sequences by keyword match

use strict;

print "Insert input file name:\n";
my $inputfile = <STDIN>;
chomp $inputfile;

print "Insert name for the output file:\n";
my $outputfile = <STDIN>;
chomp $outputfile;

open(DATA, $inputfile) || die "ERROR opening input file\n";
my @todo = <DATA>;
close(DATA);

my @selected;
my $i;

foreach my $k (@todo){
    if ($k=~ m/protamine|histone|chromatin|swi\Wsnf|ino80|chd\w|mi-
        2|centromere|dna\Wdamage|dna\Wrepair|dna\Wbinding|double\Wstran
        d\Wbreak|high\Wmobility\Wgroup|hmg[a,b,n]|nucleosom|epigenetic|
        post\Wtranslational\Wmodification|saga\W|rsc\W|nurf\W|chrac\W|a
        cf\W|rsf\W/i)  {
        push (@selected,$k);
    }
}

open(DATAOUT, ">$outputfile") || die "ERROR generating outfile\n";
print DATAOUT @selected;
close DATAOUT;


exit;
```

Figure S3. Differential expression analysis results displaying unigenes with False Discovery Rate (FDR) < 0.1.

unigenes	logFC	logCPM	LR	P-Value	FDR
XP_002587564	-10.25158	11.62690	19.27328	0.00001	0.03494
CAD79340	-10.10697	11.48231	18.91439	0.00001	0.03494
XP_786549	-9.97335	11.34872	18.58287	0.00002	0.03494
CBN81883	-9.92006	11.29544	18.45067	0.00002	0.03494
XP_615721	-9.91270	11.28809	18.43243	0.00002	0.03494
XP_002738701	-9.89490	11.27028	18.38826	0.00002	0.03494
XP_002115324	-9.52408	10.89956	17.46895	0.00003	0.03649
XP_003460288	-9.47698	10.85247	17.35226	0.00003	0.03649
XP_002737341	-9.43652	10.81202	17.25203	0.00003	0.03649
XP_003417531	-9.31457	10.69011	16.95000	0.00004	0.03649
XP_003373959	-9.28753	10.66308	16.88304	0.00004	0.03649
EFN87950	-9.24599	10.62155	16.78021	0.00004	0.03649
XP_001973709	-9.21523	10.59080	16.70407	0.00004	0.03649
XP_002601470	-9.19839	10.57397	16.66239	0.00004	0.03649
XP_002732922	9.22415	10.86852	16.56410	0.00005	0.03649
XP_002605693	-8.97367	10.34933	16.10643	0.00006	0.03807
XP_002741302	-8.95660	10.33227	16.06424	0.00006	0.03807
XP_002434442	-8.95374	10.32941	16.05715	0.00006	0.03807
XP_002609450	-8.94511	10.32078	16.03583	0.00006	0.03807
XP_002599067	-8.83425	10.20997	15.76179	0.00007	0.04131
XP_002585781	-8.80594	10.18167	15.69185	0.00007	0.04131
XP_003742476	-8.72762	10.10339	15.49839	0.00008	0.04368
XP_785416	8.74077	10.38605	15.37011	0.00009	0.04471
XP_782273	-8.55317	9.92904	15.06775	0.00010	0.04538
YP_001474807	-8.51870	9.89459	14.98272	0.00011	0.04538
AAI61151	-8.47543	9.85133	14.87597	0.00011	0.04538
XP_003727072	-8.45936	9.83528	14.83636	0.00012	0.04538
XP_003447001	8.51848	10.16428	14.82215	0.00012	0.04538
BAC39029	-8.43081	9.80674	14.76596	0.00012	0.04538
AES07023	-8.42255	9.79849	14.74559	0.00012	0.04538
XP_003208890	-8.40588	9.78183	14.70450	0.00013	0.04538
XP_002603759	-8.38051	9.75648	14.64197	0.00013	0.04538
XP_782949	-8.31952	9.69553	14.49170	0.00014	0.04538
ABO61331	-8.29257	9.66859	14.42531	0.00015	0.04538
XP_002613292	8.35630	10.00253	14.42289	0.00015	0.04538
EFW45850	8.31568	9.96203	14.32298	0.00015	0.04538
XP_001631596	-7.91962	11.04169	14.30354	0.00016	0.04538
H2A0L8	-8.23240	9.60846	14.27713	0.00016	0.04538
NP_001106507	8.29194	9.93836	14.26461	0.00016	0.04538
XP_002597426	8.29194	9.93836	14.26461	0.00016	0.04538

XP_003126347	-8.20857	9.58465	14.21847	0.00016	0.04538
XP_426008	-8.20376	9.57984	14.20662	0.00016	0.04538
XP_001748929	-8.14972	9.52584	14.07364	0.00018	0.04548
XP_002600133	8.20565	9.85234	14.05251	0.00018	0.04548
XP_003222256	-8.11425	9.49040	13.98638	0.00018	0.04548
YP_721699	-8.09878	9.47494	13.94833	0.00019	0.04548
XP_003728402	8.13737	9.78428	13.88479	0.00019	0.04548
XP_002739711	-8.06201	9.43820	13.85793	0.00020	0.04548
BAL27710	-8.05134	9.42753	13.83168	0.00020	0.04548
EGI61398	-8.04597	9.42217	13.81848	0.00020	0.04548
XP_003741028	-8.04597	9.42217	13.81848	0.00020	0.04548
XP_001625301	-8.02974	9.40595	13.77859	0.00021	0.04548
XP_002606494	-8.02429	9.40051	13.76520	0.00021	0.04548
XP_003701050	-7.99673	9.37297	13.69746	0.00021	0.04558
XP_003690478	8.05868	9.70585	13.69163	0.00022	0.04558
XP_002741848	8.04455	9.69177	13.65695	0.00022	0.04559
XP_002736149	8.00495	9.65231	13.55982	0.00023	0.04559
BAB23185	-7.93417	9.31046	13.54379	0.00023	0.04559
NP_502235	7.99763	9.64502	13.54187	0.00023	0.04559
EFX84936	-7.91663	9.29294	13.50071	0.00024	0.04559
XP_002740733	7.97917	9.62662	13.49661	0.00024	0.04559
XP_003727690	7.91458	9.56228	13.33831	0.00026	0.04694
XP_002130810	-7.85042	9.22678	13.33818	0.00026	0.04694
XP_002602259	-7.85042	9.22678	13.33818	0.00026	0.04694
XP_002427821	-7.84424	9.22062	13.32303	0.00026	0.04694
GAA33255	7.89107	9.53886	13.28073	0.00027	0.04728
XP_002613584	7.85913	9.50704	13.20249	0.00028	0.04792
ABO33165	-7.79389	9.17031	13.19950	0.00028	0.04792
CAJ82869	-7.76803	9.14448	13.13611	0.00029	0.04885
ZP_01262383	-7.74834	9.12480	13.08782	0.00030	0.04906
XP_002599303	7.79303	9.44121	13.04070	0.00030	0.04906
XP_002590998	-7.72836	9.10485	13.03886	0.00031	0.04906
AFI39909	-7.72165	9.09814	13.02240	0.00031	0.04906
XP_002608213	7.75447	9.40282	12.94639	0.00032	0.04993
XP_003223296	-7.68757	9.06410	12.93892	0.00032	0.04993
XP_002613213	7.73700	9.38542	12.90366	0.00033	0.05021
AEV89778	-7.63848	9.01506	12.81868	0.00034	0.05155
XP_003696069	-7.62414	9.00073	12.78357	0.00035	0.05155
XP_002604184	-7.61692	8.99352	12.76589	0.00035	0.05155
GAA31306	7.66952	9.31824	12.73876	0.00036	0.05155
XP_313650	7.66491	9.31365	12.72749	0.00036	0.05155
XP_002613081	-7.59503	8.97165	12.71231	0.00036	0.05155
XP_001843141	-7.58024	8.95689	12.67615	0.00037	0.05178
XP_421979	7.63692	9.28579	12.65913	0.00037	0.05178

EFN85665	-7.53497	8.91167	12.56541	0.00039	0.05356
EFA84418	-7.51956	8.89627	12.52773	0.00040	0.05356
XP_002603588	-7.51179	8.88852	12.50873	0.00041	0.05356
XP_003728822	-7.51179	8.88852	12.50873	0.00041	0.05356
EFA11608	-7.49613	8.87287	12.47045	0.00041	0.05398
XP_002732097	-7.48824	8.86499	12.45115	0.00042	0.05398
XP_786504	-7.48030	8.85706	12.43175	0.00042	0.05398
XP_002731879	-7.43993	8.81674	12.33313	0.00044	0.05566
XP_001631483	-7.42346	8.80029	12.29292	0.00045	0.05566
XP_002731704	-7.42346	8.80029	12.29292	0.00045	0.05566
XP_002731924	7.47245	9.12213	12.25795	0.00046	0.05566
XP_002596309	-7.40681	8.78366	12.25224	0.00046	0.05566
XP_001362648	7.46185	9.11158	12.23213	0.00047	0.05566
XP_001663053	-7.39841	8.77527	12.23173	0.00047	0.05566
XP_003751729	7.44581	9.09563	12.19305	0.00048	0.05566
GAA29877	-7.00087	10.68109	12.18212	0.00048	0.05566
Q05025	7.44042	9.09027	12.17993	0.00048	0.05566
AAF09840	-7.36430	8.74121	12.14850	0.00049	0.05583
XP_781514	-7.35565	8.73257	12.12739	0.00050	0.05583
XP_002600118	7.40213	9.05218	12.08672	0.00051	0.05583
XP_002742050	-7.32937	8.70633	12.06329	0.00051	0.05583
EFX76362	7.39101	9.04112	12.05964	0.00052	0.05583
XP_003480922	7.39101	9.04112	12.05964	0.00052	0.05583
XP_002414307	-7.32051	8.69747	12.04167	0.00052	0.05583
YP_131523	-7.31159	8.68856	12.01992	0.00053	0.05583
XP_001023282	-7.30261	8.67960	11.99803	0.00053	0.05583
XP_003401618	-7.30261	8.67960	11.99803	0.00053	0.05583
XP_002609208	7.34561	8.99598	11.94922	0.00055	0.05630
XP_003646937	7.34561	8.99598	11.94922	0.00055	0.05630
NP_001232024	-7.26613	8.64318	11.90914	0.00056	0.05702
XP_001635351	-7.22871	8.60581	11.81798	0.00059	0.05936
XP_002601497	-7.02918	9.24389	11.78751	0.00060	0.05982
XP_002591663	7.25030	8.90122	11.71764	0.00062	0.06110
AEE62746	7.24413	8.89509	11.70266	0.00062	0.06110
XP_002708436	-7.18053	8.55770	11.70068	0.00062	0.06110
ZP_08909002	-7.14079	8.51802	11.60401	0.00066	0.06356
XP_002939441	7.20018	8.85141	11.59600	0.00066	0.06356
XP_002607896	7.19379	8.84506	11.58050	0.00067	0.06357
CAB82366	-7.12050	8.49776	11.55467	0.00068	0.06394
XP_002609379	7.17445	8.82584	11.53360	0.00068	0.06414
XP_002435427	-7.09992	8.47722	11.50466	0.00069	0.06421
XP_002740460	7.16141	8.81288	11.50199	0.00070	0.06421
XP_002606415	6.72334	10.72772	11.47483	0.00071	0.06426
XP_002668154	7.14825	8.79981	11.47009	0.00071	0.06426

XP_001641836	-7.07905	8.45637	11.45393	0.00071	0.06426
XP_002416575	7.13497	8.78661	11.43792	0.00072	0.06426
XP_003725380	-7.06850	8.44584	11.42830	0.00072	0.06426
ADI24338	7.11482	8.76659	11.38910	0.00074	0.06513
XP_003388665	7.10122	8.75309	11.35618	0.00075	0.06545
XP_002160973	-7.02550	8.40291	11.32389	0.00077	0.06545
XP_003705881	-7.02550	8.40291	11.32389	0.00077	0.06545
XP_002594308	7.08059	8.73260	11.30624	0.00077	0.06545
XP_002943965	-7.01455	8.39198	11.29731	0.00078	0.06545
XP_003212697	-7.01455	8.39198	11.29731	0.00078	0.06545
XP_790859	-6.56744	11.21975	11.27431	0.00079	0.06546
XP_635792	-7.00351	8.38096	11.27053	0.00079	0.06546
XP_002738133	7.05262	8.70481	11.23854	0.00080	0.06580
CBJ25740	7.04554	8.69778	11.22142	0.00081	0.06580
XP_003202487	7.04554	8.69778	11.22142	0.00081	0.06580
XP_001365438	-6.96988	8.34740	11.18897	0.00082	0.06650
XP_002602545	-6.95850	8.33603	11.16136	0.00084	0.06656
XP_002604134	-6.95850	8.33603	11.16136	0.00084	0.06656
ABY20700	-6.94702	8.32458	11.13354	0.00085	0.06656
XP_003747201	-6.93546	8.31303	11.10551	0.00086	0.06656
ZP_01262732	-6.93546	8.31303	11.10551	0.00086	0.06656
XP_002740538	6.99498	8.64758	11.09920	0.00086	0.06656
XP_798461	6.99498	8.64758	11.09920	0.00086	0.06656
XP_002590771	6.98020	8.63291	11.06350	0.00088	0.06681
NP_001082876	-6.91204	8.28966	11.04877	0.00089	0.06681
AAB66556	-6.90019	8.27783	11.02007	0.00090	0.06681
CAG09487	-6.90019	8.27783	11.02007	0.00090	0.06681
XP_002427858	-6.90019	8.27783	11.02007	0.00090	0.06681
XP_783551	-6.90019	8.27783	11.02007	0.00090	0.06681
XP_001603746	-6.88824	8.26590	10.99113	0.00092	0.06743
XP_003449362	-6.87619	8.25387	10.96195	0.00093	0.06807
EHB08796	-6.86403	8.24174	10.93254	0.00094	0.06813
XP_002732889	6.91954	8.57269	10.91706	0.00095	0.06813
XP_968505	6.91954	8.57269	10.91706	0.00095	0.06813
ACO51858	-6.85178	8.22951	10.90289	0.00096	0.06813
BAK09601	-6.85178	8.22951	10.90289	0.00096	0.06813
XP_002602771	6.90397	8.55724	10.87949	0.00097	0.06840
XP_798483	-6.83942	8.21718	10.87298	0.00098	0.06840
XP_003221019	-6.82695	8.20473	10.84283	0.00099	0.06911
ZP_01254585	-6.81437	8.19218	10.81242	0.00101	0.06984
EHJ68398	-6.80169	8.17952	10.78175	0.00103	0.07042
XP_001842802	6.85621	8.50985	10.76438	0.00103	0.07042
XP_002587109	6.85621	8.50985	10.76438	0.00103	0.07042
NP_001072851	-6.78889	8.16675	10.75082	0.00104	0.07052

XP_794070	-6.77598	8.15386	10.71962	0.00106	0.07131
AEK81538	-6.76295	8.14086	10.68815	0.00108	0.07170
XP_002433513	-6.76295	8.14086	10.68815	0.00108	0.07170
AFE71369	-6.73653	8.11450	10.62436	0.00112	0.07338
XP_002739967	-6.73653	8.11450	10.62436	0.00112	0.07338
XP_002598499	6.78997	8.44415	10.60493	0.00113	0.07374
BAE39186	-6.72313	8.10113	10.59204	0.00114	0.07375
AAB33368	-6.70962	8.08764	10.55942	0.00116	0.07375
AAL99291	-6.70962	8.08764	10.55942	0.00116	0.07375
XP_002596753	-6.70962	8.08764	10.55942	0.00116	0.07375
AAH72205	6.76433	8.41872	10.54326	0.00117	0.07375
XP_002603175	6.76433	8.41872	10.54326	0.00117	0.07375
XP_001637862	6.75568	8.41014	10.52247	0.00118	0.07395
ABO61332	-6.68219	8.06028	10.49328	0.00120	0.07395
EGT39498	-6.68219	8.06028	10.49328	0.00120	0.07395
XP_003700634	-6.68219	8.06028	10.49328	0.00120	0.07395
XP_002730788	6.73822	8.39283	10.48051	0.00121	0.07395
ACN91289	-6.66828	8.04640	10.45975	0.00122	0.07395
GAA48059	-6.66828	8.04640	10.45975	0.00122	0.07395
XP_002664535	-6.66828	8.04640	10.45975	0.00122	0.07395
CBL22496	-6.64006	8.01824	10.39174	0.00127	0.07593
XP_002598321	-6.64006	8.01824	10.39174	0.00127	0.07593
XP_001633780	-6.62573	8.00395	10.35724	0.00129	0.07696
BAE90615	-6.61126	7.98951	10.32241	0.00131	0.07721
EDL08213	-6.59665	7.97493	10.28724	0.00134	0.07721
XP_001850184	-6.59665	7.97493	10.28724	0.00134	0.07721
XP_002732920	-6.59665	7.97493	10.28724	0.00134	0.07721
XP_003241032	-6.59665	7.97493	10.28724	0.00134	0.07721
XP_003382138	6.63823	8.29372	10.24058	0.00137	0.07721
XP_002741129	6.62879	8.28436	10.21795	0.00139	0.07721
XP_002193200	-6.56697	7.94533	10.21584	0.00139	0.07721
XP_003724601	-6.56697	7.94533	10.21584	0.00139	0.07721
XP_001301486	6.61929	8.27495	10.19518	0.00141	0.07721
AAX26400	-6.55190	7.93029	10.17960	0.00142	0.07721
CAB70731	-6.55190	7.93029	10.17960	0.00142	0.07721
CAH90435	-6.55190	7.93029	10.17960	0.00142	0.07721
EFX69098	-6.55190	7.93029	10.17960	0.00142	0.07721
XP_003291621	-6.55190	7.93029	10.17960	0.00142	0.07721
XP_003707588	-6.55190	7.93029	10.17960	0.00142	0.07721
XP_003723792	-6.55190	7.93029	10.17960	0.00142	0.07721
EFN60512	6.60972	8.26547	10.17227	0.00143	0.07721
AAH89352	-6.53667	7.91510	10.14300	0.00145	0.07721
CAL49346	-6.53667	7.91510	10.14300	0.00145	0.07721
XP_002613975	-6.53667	7.91510	10.14300	0.00145	0.07721

XP_002738101	-6.53667	7.91510	10.14300	0.00145	0.07721
AAN08149	-6.52127	7.89974	10.10602	0.00148	0.07721
XP_003386162	-6.52127	7.89974	10.10602	0.00148	0.07721
EAY91070	6.58063	8.23665	10.10263	0.00148	0.07721
NP_001036021	6.58063	8.23665	10.10263	0.00148	0.07721
XP_002728784	6.58063	8.23665	10.10263	0.00148	0.07721
CAJ83444	6.57080	8.22691	10.07911	0.00150	0.07721
CBQ69465	-6.50571	7.88422	10.06865	0.00151	0.07721
XP_001623551	6.56090	8.21711	10.05544	0.00152	0.07721
XP_002612285	6.55094	8.20724	10.03161	0.00154	0.07721
ZP_09111650	6.55094	8.20724	10.03161	0.00154	0.07721
AAX84973	-6.48999	7.86853	10.03089	0.00154	0.07721
AEP17623	-6.48999	7.86853	10.03089	0.00154	0.07721
XP_001807104	-6.48999	7.86853	10.03089	0.00154	0.07721
XP_002122175	-6.48999	7.86853	10.03089	0.00154	0.07721
XP_002589575	-6.48999	7.86853	10.03089	0.00154	0.07721
XP_003387894	6.54090	8.19730	10.00762	0.00156	0.07749
NP_958889	-6.47408	7.85267	9.99273	0.00157	0.07749
XP_002404411	-6.47408	7.85267	9.99273	0.00157	0.07749
XP_780780	-6.47408	7.85267	9.99273	0.00157	0.07749
AAK95589	6.53080	8.18730	9.98347	0.00158	0.07756
XP_002608858	6.52062	8.17722	9.95916	0.00160	0.07826
BAE22050	-6.44174	7.82042	9.91517	0.00164	0.07915
XP_002737009	-6.44174	7.82042	9.91517	0.00164	0.07915
XP_002738835	-6.44174	7.82042	9.91517	0.00164	0.07915
CAX74044	-6.42530	7.80401	9.87575	0.00167	0.08020
XP_002732582	-6.42530	7.80401	9.87575	0.00167	0.08020
AAL37183	-6.40866	7.78742	9.83590	0.00171	0.08034
ADR79275	-6.40866	7.78742	9.83590	0.00171	0.08034
XP_002739336	-6.40866	7.78742	9.83590	0.00171	0.08034
YP_720839	-6.40866	7.78742	9.83590	0.00171	0.08034
XP_001625155	6.46864	8.12576	9.83506	0.00171	0.08034
CBY06820	6.45801	8.11524	9.80972	0.00174	0.08067
NP_001191540	-6.39183	7.77064	9.79560	0.00175	0.08067
XP_002600408	-6.39183	7.77064	9.79560	0.00175	0.08067
XP_001373115	6.44731	8.10465	9.78420	0.00176	0.08067
ABO26647	-6.37480	7.75366	9.75484	0.00179	0.08067
BAC34934	-6.37480	7.75366	9.75484	0.00179	0.08067
CAZ27720	-6.37480	7.75366	9.75484	0.00179	0.08067
CCD21012	-6.37480	7.75366	9.75484	0.00179	0.08067
XP_001514950	-6.37480	7.75366	9.75484	0.00179	0.08067
XP_697184	-6.37480	7.75366	9.75484	0.00179	0.08067
XP_002198814	6.42566	8.08322	9.73260	0.00181	0.08102
XP_002734374	6.42566	8.08322	9.73260	0.00181	0.08102

BAK40203	-6.35757	7.73648	9.71361	0.00183	0.08155
AEN04481	6.40368	8.06148	9.68025	0.00186	0.08155
ACE75736	-6.34013	7.71909	9.67191	0.00187	0.08155
BAC06836	-6.34013	7.71909	9.67191	0.00187	0.08155
CAH04106	-6.34013	7.71909	9.67191	0.00187	0.08155
CCA37660	-6.34013	7.71909	9.67191	0.00187	0.08155
XP_002425888	-6.34013	7.71909	9.67191	0.00187	0.08155
AAH08024	-6.32247	7.70148	9.62971	0.00191	0.08263
XP_780658	-6.32247	7.70148	9.62971	0.00191	0.08263
XP_002607850	6.38136	8.03940	9.62713	0.00192	0.08263
XP_422162	-6.30460	7.68366	9.58702	0.00196	0.08384
XP_800195	-6.30460	7.68366	9.58702	0.00196	0.08384
XP_002605359	6.35869	8.01697	9.57321	0.00197	0.08385
XP_002736555	6.35869	8.01697	9.57321	0.00197	0.08385
XP_002401365	-6.28650	7.66562	9.54381	0.00201	0.08459
XP_002425306	-6.28650	7.66562	9.54381	0.00201	0.08459
AEO32528	-6.26818	7.64735	9.50007	0.00205	0.08509
BAC32100	-6.26818	7.64735	9.50007	0.00205	0.08509
EFN88349	-6.26818	7.64735	9.50007	0.00205	0.08509
XP_782331	-6.26818	7.64735	9.50007	0.00205	0.08509
ZP_05502520	-6.26818	7.64735	9.50007	0.00205	0.08509
XP_002735987	6.32400	7.98267	9.49079	0.00207	0.08522
XP_002594508	-6.24961	7.62885	9.45580	0.00210	0.08576
EFX83224	6.30040	7.95934	9.43476	0.00213	0.08576
XP_002121660	6.30040	7.95934	9.43476	0.00213	0.08576
NP_001191543	-6.23081	7.61010	9.41097	0.00216	0.08576
XP_002609620	-6.23081	7.61010	9.41097	0.00216	0.08576
YP_001999719	-6.23081	7.61010	9.41097	0.00216	0.08576
XP_002942405	5.84851	10.07905	9.40674	0.00216	0.08576
XP_001519063	6.27641	7.93562	9.37786	0.00220	0.08576
XP_002600359	6.27641	7.93562	9.37786	0.00220	0.08576
XP_003726941	6.27641	7.93562	9.37786	0.00220	0.08576
AAH90296	-6.21176	7.59111	9.36558	0.00221	0.08576
EFA01267	-6.21176	7.59111	9.36558	0.00221	0.08576
XP_001516235	-6.21176	7.59111	9.36558	0.00221	0.08576
XP_002599429	-6.21176	7.59111	9.36558	0.00221	0.08576
XP_002601662	-6.21176	7.59111	9.36558	0.00221	0.08576
XP_002741470	-6.21176	7.59111	9.36558	0.00221	0.08576
XP_003229251	-6.21176	7.59111	9.36558	0.00221	0.08576
ZP_04921972	-6.21176	7.59111	9.36558	0.00221	0.08576
EFR23189	-6.19245	7.57186	9.31961	0.00227	0.08622
XP_001516447	-6.19245	7.57186	9.31961	0.00227	0.08622
XP_002592002	-6.19245	7.57186	9.31961	0.00227	0.08622
XP_002600258	-6.19245	7.57186	9.31961	0.00227	0.08622

ZP_01066272	-6.19245	7.57186	9.31961	0.00227	0.08622
ZP_06300713	-6.19245	7.57186	9.31961	0.00227	0.08622
XP_001600879	-6.17288	7.55236	9.27304	0.00233	0.08699
XP_002611407	-6.17288	7.55236	9.27304	0.00233	0.08699
XP_002738916	-6.17288	7.55236	9.27304	0.00233	0.08699
XP_002741986	6.22720	7.88699	9.26126	0.00234	0.08699
ABY75514	5.75227	10.33727	9.23712	0.00237	0.08699
CCF33422	6.21463	7.87457	9.23151	0.00238	0.08699
XP_002592165	6.21463	7.87457	9.23151	0.00238	0.08699
CAL49314	-6.15304	7.53258	9.22586	0.00239	0.08699
XP_001633753	-6.15304	7.53258	9.22586	0.00239	0.08699
XP_002738829	-6.15304	7.53258	9.22586	0.00239	0.08699
ADK38674	-6.13292	7.51254	9.17805	0.00245	0.08699
XP_002732836	-6.13292	7.51254	9.17805	0.00245	0.08699
XP_002740852	-6.13292	7.51254	9.17805	0.00245	0.08699
AAH59873	-6.11252	7.49221	9.12960	0.00252	0.08699
AAH95783	-6.11252	7.49221	9.12960	0.00252	0.08699
EHJ24574	-6.11252	7.49221	9.12960	0.00252	0.08699
XP_001632062	-6.11252	7.49221	9.12960	0.00252	0.08699
XP_002405655	-6.11252	7.49221	9.12960	0.00252	0.08699
XP_002605874	-6.11252	7.49221	9.12960	0.00252	0.08699
XP_002607042	-6.11252	7.49221	9.12960	0.00252	0.08699
XP_002608924	-6.11252	7.49221	9.12960	0.00252	0.08699
XP_002643989	-6.11252	7.49221	9.12960	0.00252	0.08699
EFZ12036	-6.09183	7.47159	9.08049	0.00258	0.08699
EGI63691	-6.09183	7.47159	9.08049	0.00258	0.08699
XP_002735038	-6.09183	7.47159	9.08049	0.00258	0.08699
YP_003387842	-6.09183	7.47159	9.08049	0.00258	0.08699
XP_002613350	6.15009	7.81082	9.07895	0.00259	0.08699
XP_003206645	6.15009	7.81082	9.07895	0.00259	0.08699
XP_001344731	6.13683	7.79773	9.04764	0.00263	0.08699
XP_003505948	6.13683	7.79773	9.04764	0.00263	0.08699
XP_002739316	-5.65210	10.05969	9.03614	0.00265	0.08699
AAN40846	-6.07084	7.45067	9.03069	0.00265	0.08699
AEF33410	-6.07084	7.45067	9.03069	0.00265	0.08699
AEO33510	-6.07084	7.45067	9.03069	0.00265	0.08699
EHJ71735	-6.07084	7.45067	9.03069	0.00265	0.08699
XP_003724812	-6.07084	7.45067	9.03069	0.00265	0.08699
XP_647665	-6.07084	7.45067	9.03069	0.00265	0.08699
XP_003452834	5.68862	9.92200	9.02525	0.00266	0.08699
BAE22065	6.12344	7.78451	9.01606	0.00268	0.08699
ABX82529	-6.04953	7.42944	8.98020	0.00273	0.08699
XP_001946467	-6.04953	7.42944	8.98020	0.00273	0.08699
XP_002607725	-6.04953	7.42944	8.98020	0.00273	0.08699

XP_002611765	-6.04953	7.42944	8.98020	0.00273	0.08699
XP_002738623	-6.04953	7.42944	8.98020	0.00273	0.08699
XP_003262676	-6.04953	7.42944	8.98020	0.00273	0.08699
XP_003445121	-6.04953	7.42944	8.98020	0.00273	0.08699
XP_001603629	6.09629	7.75771	8.95206	0.00277	0.08699
XP_003391145	5.72988	9.18767	8.93806	0.00279	0.08699
XP_003413538	-5.56270	11.15289	8.92922	0.00281	0.08699
EFN68572	-6.02791	7.40790	8.92898	0.00281	0.08699
NP_083025	-6.02791	7.40790	8.92898	0.00281	0.08699
XP_002408245	-6.02791	7.40790	8.92898	0.00281	0.08699
XP_002591304	-6.02791	7.40790	8.92898	0.00281	0.08699
XP_002824001	-6.02791	7.40790	8.92898	0.00281	0.08699
XP_003445054	-6.02791	7.40790	8.92898	0.00281	0.08699
AAM22012	6.08252	7.74413	8.91962	0.00282	0.08699
YP_002840971	6.08252	7.74413	8.91962	0.00282	0.08699
BAE94191	6.06862	7.73041	8.88689	0.00287	0.08699
EGI62872	6.06862	7.73041	8.88689	0.00287	0.08699
XP_001634991	-6.00596	7.38603	8.87703	0.00289	0.08699
XP_002594881	-6.00596	7.38603	8.87703	0.00289	0.08699
XP_002604367	-6.00596	7.38603	8.87703	0.00289	0.08699
XP_002610657	-6.00596	7.38603	8.87703	0.00289	0.08699
XP_002736079	-6.00596	7.38603	8.87703	0.00289	0.08699
XP_003149509	-6.00596	7.38603	8.87703	0.00289	0.08699
XP_003443302	-6.00596	7.38603	8.87703	0.00289	0.08699
XP_003704298	-6.00596	7.38603	8.87703	0.00289	0.08699
XP_793640	-6.00596	7.38603	8.87703	0.00289	0.08699
1713406B	-5.98367	7.36382	8.82432	0.00297	0.08699
EFX90440	-5.98367	7.36382	8.82432	0.00297	0.08699
XP_001607321	-5.98367	7.36382	8.82432	0.00297	0.08699
XP_002424869	-5.98367	7.36382	8.82432	0.00297	0.08699
XP_002430886	-5.98367	7.36382	8.82432	0.00297	0.08699
XP_002590067	-5.98367	7.36382	8.82432	0.00297	0.08699
XP_002608259	-5.98367	7.36382	8.82432	0.00297	0.08699
XP_003379741	-5.98367	7.36382	8.82432	0.00297	0.08699
XP_003495220	-5.98367	7.36382	8.82432	0.00297	0.08699
XP_003706807	-5.98367	7.36382	8.82432	0.00297	0.08699
XP_003724102	-5.98367	7.36382	8.82432	0.00297	0.08699
XP_003728083	-5.98367	7.36382	8.82432	0.00297	0.08699
EFA75352	6.04041	7.70257	8.82052	0.00298	0.08699
CAF87168	-5.96103	7.34127	8.77082	0.00306	0.08699
CCD82066	-5.96103	7.34127	8.77082	0.00306	0.08699
EHJ70170	-5.96103	7.34127	8.77082	0.00306	0.08699
P07201	-5.96103	7.34127	8.77082	0.00306	0.08699
XP_003223024	-5.96103	7.34127	8.77082	0.00306	0.08699

XP_003276168	-5.96103	7.34127	8.77082	0.00306	0.08699
XP_002735518	6.01163	7.67419	8.75289	0.00309	0.08699
XP_003706805	6.01163	7.67419	8.75289	0.00309	0.08699
XP_003727906	5.99703	7.65979	8.71860	0.00315	0.08699
AEO35054	-5.93802	7.31836	8.71651	0.00315	0.08699
CAI46182	-5.93802	7.31836	8.71651	0.00315	0.08699
CAJ30045	-5.93802	7.31836	8.71651	0.00315	0.08699
EFA04949	-5.93802	7.31836	8.71651	0.00315	0.08699
EFX88629	-5.93802	7.31836	8.71651	0.00315	0.08699
EFX89033	-5.93802	7.31836	8.71651	0.00315	0.08699
XP_002124863	-5.93802	7.31836	8.71651	0.00315	0.08699
XP_002597968	-5.93802	7.31836	8.71651	0.00315	0.08699
XP_002735223	-5.93802	7.31836	8.71651	0.00315	0.08699
XP_003255664	-5.93802	7.31836	8.71651	0.00315	0.08699
XP_003448039	-5.93802	7.31836	8.71651	0.00315	0.08699
XP_003448299	-5.93802	7.31836	8.71651	0.00315	0.08699
XP_003693223	-5.93802	7.31836	8.71651	0.00315	0.08699
XP_001637561	5.98227	7.64524	8.68398	0.00321	0.08699
XP_002939897	5.98227	7.64524	8.68398	0.00321	0.08699
AAK72969	-5.91465	7.29508	8.66137	0.00325	0.08699
AAV69062	-5.91465	7.29508	8.66137	0.00325	0.08699
CAI40491	-5.91465	7.29508	8.66137	0.00325	0.08699
EFW20245	-5.91465	7.29508	8.66137	0.00325	0.08699
XP_002602361	-5.91465	7.29508	8.66137	0.00325	0.08699
XP_003728064	-5.91465	7.29508	8.66137	0.00325	0.08699
NP_001170929	5.96737	7.63054	8.64901	0.00327	0.08699
AAK12359	5.95230	7.61569	8.61371	0.00334	0.08699
XP_002606412	5.95230	7.61569	8.61371	0.00334	0.08699
XP_003209223	5.95230	7.61569	8.61371	0.00334	0.08699
XP_003364926	5.95230	7.61569	8.61371	0.00334	0.08699
AEV53960	-5.89089	7.27142	8.60537	0.00335	0.08699
CAJ83480	-5.89089	7.27142	8.60537	0.00335	0.08699
EFX69526	-5.89089	7.27142	8.60537	0.00335	0.08699
XP_001017101	-5.89089	7.27142	8.60537	0.00335	0.08699
XP_001508218	-5.89089	7.27142	8.60537	0.00335	0.08699
XP_002262216	-5.89089	7.27142	8.60537	0.00335	0.08699
XP_002595411	-5.89089	7.27142	8.60537	0.00335	0.08699
XP_002735625	-5.89089	7.27142	8.60537	0.00335	0.08699
XP_002815001	-5.89089	7.27142	8.60537	0.00335	0.08699
XP_002940311	-5.89089	7.27142	8.60537	0.00335	0.08699
XP_003428681	-5.89089	7.27142	8.60537	0.00335	0.08699
XP_789620	-5.89089	7.27142	8.60537	0.00335	0.08699
NP_001191667	5.93708	7.60069	8.57805	0.00340	0.08699
XP_002046731	5.93708	7.60069	8.57805	0.00340	0.08699

XP_784034	5.93708	7.60069	8.57805	0.00340	0.08699
ACN58711	-5.86673	7.24736	8.54848	0.00346	0.08699
CAJ26346	-5.86673	7.24736	8.54848	0.00346	0.08699
XP_001008643	-5.86673	7.24736	8.54848	0.00346	0.08699
XP_001426760	-5.86673	7.24736	8.54848	0.00346	0.08699
XP_002738904	-5.86673	7.24736	8.54848	0.00346	0.08699
XP_002752766	-5.86673	7.24736	8.54848	0.00346	0.08699
XP_003390327	-5.86673	7.24736	8.54848	0.00346	0.08699
XP_786649	-5.86673	7.24736	8.54848	0.00346	0.08699
XP_975362	-5.86673	7.24736	8.54848	0.00346	0.08699
YP_004179856	-5.86673	7.24736	8.54848	0.00346	0.08699
ZP_09483044	-5.86673	7.24736	8.54848	0.00346	0.08699
AAR39412	5.92170	7.58553	8.54204	0.00347	0.08699
XP_003377920	5.92170	7.58553	8.54204	0.00347	0.08699
XP_003723764	5.92170	7.58553	8.54204	0.00347	0.08699
NP_001155276	5.90615	7.57021	8.50567	0.00354	0.08699
XP_002115163	5.90615	7.57021	8.50567	0.00354	0.08699
XP_002193920	5.90615	7.57021	8.50567	0.00354	0.08699
XP_002596572	5.90615	7.57021	8.50567	0.00354	0.08699
XP_003425652	5.90615	7.57021	8.50567	0.00354	0.08699
AAY86961	-5.84216	7.22290	8.49068	0.00357	0.08699
EHH55556	-5.84216	7.22290	8.49068	0.00357	0.08699
XP_002070310	-5.84216	7.22290	8.49068	0.00357	0.08699
XP_002169253	-5.84216	7.22290	8.49068	0.00357	0.08699
XP_002588513	-5.84216	7.22290	8.49068	0.00357	0.08699
XP_002611352	-5.84216	7.22290	8.49068	0.00357	0.08699
XP_002698598	-5.84216	7.22290	8.49068	0.00357	0.08699
XP_536929	-5.84216	7.22290	8.49068	0.00357	0.08699
XP_002400528	-5.65648	7.88110	8.48731	0.00358	0.08699
AAH24689	5.89043	7.55472	8.46892	0.00361	0.08699
AAI69208	5.89043	7.55472	8.46892	0.00361	0.08699
XP_002610105	5.89043	7.55472	8.46892	0.00361	0.08699
XP_003217401	5.89043	7.55472	8.46892	0.00361	0.08699
AAT09325	-5.81717	7.19802	8.43193	0.00369	0.08699
BAA78421	-5.81717	7.19802	8.43193	0.00369	0.08699
CAX33834	-5.81717	7.19802	8.43193	0.00369	0.08699
EFN66701	-5.81717	7.19802	8.43193	0.00369	0.08699
EFN68468	-5.81717	7.19802	8.43193	0.00369	0.08699
EGI59904	-5.81717	7.19802	8.43193	0.00369	0.08699
XP_001623622	-5.81717	7.19802	8.43193	0.00369	0.08699
XP_001637157	-5.81717	7.19802	8.43193	0.00369	0.08699
XP_001992750	-5.81717	7.19802	8.43193	0.00369	0.08699
XP_002399509	-5.81717	7.19802	8.43193	0.00369	0.08699
XP_002735917	-5.81717	7.19802	8.43193	0.00369	0.08699

XP_002742001	-5.81717	7.19802	8.43193	0.00369	0.08699
XP_785443	-5.81717	7.19802	8.43193	0.00369	0.08699
XP_001628920	5.87453	7.53907	8.43179	0.00369	0.08699
XP_001640068	5.87453	7.53907	8.43179	0.00369	0.08699
XP_003199319	5.87453	7.53907	8.43179	0.00369	0.08699
XP_002742380	5.65760	8.18983	8.41661	0.00372	0.08699
CAF21863	5.85847	7.52324	8.39427	0.00376	0.08699
EGR50668	5.85847	7.52324	8.39427	0.00376	0.08699
XP_002608670	5.85847	7.52324	8.39427	0.00376	0.08699
XP_002612503	5.85847	7.52324	8.39427	0.00376	0.08699
XP_002732400	5.85847	7.52324	8.39427	0.00376	0.08699
XP_419581	5.85847	7.52324	8.39427	0.00376	0.08699
AEO34023	-5.79173	7.17269	8.37221	0.00381	0.08699
BAE39153	-5.79173	7.17269	8.37221	0.00381	0.08699
CBK21306	-5.79173	7.17269	8.37221	0.00381	0.08699
EAW94898	-5.79173	7.17269	8.37221	0.00381	0.08699
EFX67003	-5.79173	7.17269	8.37221	0.00381	0.08699
EGT56672	-5.79173	7.17269	8.37221	0.00381	0.08699
NP_001245290	-5.79173	7.17269	8.37221	0.00381	0.08699
XP_001605649	-5.79173	7.17269	8.37221	0.00381	0.08699
XP_001633418	-5.79173	7.17269	8.37221	0.00381	0.08699
XP_002427447	-5.79173	7.17269	8.37221	0.00381	0.08699
XP_002606880	-5.79173	7.17269	8.37221	0.00381	0.08699
XP_002608868	-5.79173	7.17269	8.37221	0.00381	0.08699
XP_003471921	-5.79173	7.17269	8.37221	0.00381	0.08699
XP_785456	-5.79173	7.17269	8.37221	0.00381	0.08699
XP_969024	-5.79173	7.17269	8.37221	0.00381	0.08699
XP_002736461	-5.33422	10.49879	8.35754	0.00384	0.08699
BAD99027	5.84221	7.50724	8.35636	0.00384	0.08699
XP_001635963	5.84221	7.50724	8.35636	0.00384	0.08699
XP_001652929	5.84221	7.50724	8.35636	0.00384	0.08699
XP_001989824	5.84221	7.50724	8.35636	0.00384	0.08699
XP_002588348	5.84221	7.50724	8.35636	0.00384	0.08699
XP_002933745	-5.33820	10.00903	8.32145	0.00392	0.08699
ADL62715	5.82578	7.49106	8.31804	0.00393	0.08699
EFA84096	5.82578	7.49106	8.31804	0.00393	0.08699
XP_002741344	5.82578	7.49106	8.31804	0.00393	0.08699
AAI69185	-5.76584	7.14692	8.31149	0.00394	0.08699
AAV84265	-5.76584	7.14692	8.31149	0.00394	0.08699
NP_001073671	-5.76584	7.14692	8.31149	0.00394	0.08699
XP_002162663	-5.76584	7.14692	8.31149	0.00394	0.08699
XP_002592492	-5.76584	7.14692	8.31149	0.00394	0.08699
XP_002601381	-5.76584	7.14692	8.31149	0.00394	0.08699
XP_002605750	-5.76584	7.14692	8.31149	0.00394	0.08699

XP_002612895	-5.76584	7.14692	8.31149	0.00394	0.08699
XP_002736557	-5.76584	7.14692	8.31149	0.00394	0.08699
XP_422260	-5.76584	7.14692	8.31149	0.00394	0.08699
XP_002739922	5.80915	7.47469	8.27931	0.00401	0.08783
XP_791927	-5.26332	11.75548	8.25921	0.00405	0.08783
AAV48595	-5.73947	7.12068	8.24971	0.00408	0.08783
ACH89433	-5.73947	7.12068	8.24971	0.00408	0.08783
AER98881	-5.73947	7.12068	8.24971	0.00408	0.08783
NP_957183	-5.73947	7.12068	8.24971	0.00408	0.08783
XP_002190212	-5.73947	7.12068	8.24971	0.00408	0.08783
XP_002433601	-5.73947	7.12068	8.24971	0.00408	0.08783
XP_002434360	-5.73947	7.12068	8.24971	0.00408	0.08783
XP_002597222	-5.73947	7.12068	8.24971	0.00408	0.08783
XP_002607260	-5.73947	7.12068	8.24971	0.00408	0.08783
XP_002940183	-5.73947	7.12068	8.24971	0.00408	0.08783
XP_415238	-5.73947	7.12068	8.24971	0.00408	0.08783
XP_002593922	5.79233	7.45814	8.24016	0.00410	0.08797
XP_974007	-5.54130	7.76727	8.21621	0.00415	0.08797
EDL24736	5.77532	7.44140	8.20058	0.00419	0.08797
XP_002734689	5.77532	7.44140	8.20058	0.00419	0.08797
XP_967104	5.77532	7.44140	8.20058	0.00419	0.08797
EAW75624	-5.71262	7.09395	8.18686	0.00422	0.08797
EFX83489	-5.71262	7.09395	8.18686	0.00422	0.08797
XP_001632203	-5.71262	7.09395	8.18686	0.00422	0.08797
XP_002598814	-5.71262	7.09395	8.18686	0.00422	0.08797
XP_002742202	-5.71262	7.09395	8.18686	0.00422	0.08797
XP_002413853	5.75810	7.42445	8.16055	0.00428	0.08797
XP_002593669	5.75810	7.42445	8.16055	0.00428	0.08797
XP_003391309	5.75810	7.42445	8.16055	0.00428	0.08797
2XNF_A	-5.68525	7.06672	8.12289	0.00437	0.08797
AAM91821	-5.68525	7.06672	8.12289	0.00437	0.08797
ACG75958	-5.68525	7.06672	8.12289	0.00437	0.08797
AES02438	-5.68525	7.06672	8.12289	0.00437	0.08797
AES03553	-5.68525	7.06672	8.12289	0.00437	0.08797
CAA59198	-5.68525	7.06672	8.12289	0.00437	0.08797
EFN66218	-5.68525	7.06672	8.12289	0.00437	0.08797
GAA50512	-5.68525	7.06672	8.12289	0.00437	0.08797
NP_001135486	-5.68525	7.06672	8.12289	0.00437	0.08797
NP_001164310	-5.68525	7.06672	8.12289	0.00437	0.08797
XP_001621329	-5.68525	7.06672	8.12289	0.00437	0.08797
XP_001627192	-5.68525	7.06672	8.12289	0.00437	0.08797
XP_002063027	-5.68525	7.06672	8.12289	0.00437	0.08797
XP_002613173	-5.68525	7.06672	8.12289	0.00437	0.08797
XP_002712574	-5.68525	7.06672	8.12289	0.00437	0.08797

XP_002734698	-5.68525	7.06672	8.12289	0.00437	0.08797
XP_002735688	-5.68525	7.06672	8.12289	0.00437	0.08797
XP_002942697	-5.68525	7.06672	8.12289	0.00437	0.08797
XP_003206052	-5.68525	7.06672	8.12289	0.00437	0.08797
XP_640602	-5.68525	7.06672	8.12289	0.00437	0.08797
XP_701169	-5.68525	7.06672	8.12289	0.00437	0.08797
XP_785654	-5.68525	7.06672	8.12289	0.00437	0.08797
CAA67544	5.74067	7.40731	8.12008	0.00438	0.08797
CAG04340	5.74067	7.40731	8.12008	0.00438	0.08797
NP_001079493	5.74067	7.40731	8.12008	0.00438	0.08797
AAH94402	5.72303	7.38996	8.07914	0.00448	0.08797
EFX81396	5.72303	7.38996	8.07914	0.00448	0.08797
XP_002077667	5.72303	7.38996	8.07914	0.00448	0.08797
XP_417386	5.72303	7.38996	8.07914	0.00448	0.08797
YP_006354273	5.72303	7.38996	8.07914	0.00448	0.08797
AAH94088	-5.65736	7.03897	8.05776	0.00453	0.08797
AEO34555	-5.65736	7.03897	8.05776	0.00453	0.08797
BAE35720	-5.65736	7.03897	8.05776	0.00453	0.08797
NP_998032	-5.65736	7.03897	8.05776	0.00453	0.08797
XP_001944340	-5.65736	7.03897	8.05776	0.00453	0.08797
XP_001999880	-5.65736	7.03897	8.05776	0.00453	0.08797
XP_002127989	-5.65736	7.03897	8.05776	0.00453	0.08797
XP_002197995	-5.65736	7.03897	8.05776	0.00453	0.08797
XP_002587935	-5.65736	7.03897	8.05776	0.00453	0.08797
ACQ91104	-5.47110	7.69795	8.05161	0.00455	0.08797
NP_001019499	-5.47110	7.69795	8.05161	0.00455	0.08797
NP_001133096	-5.47110	7.69795	8.05161	0.00455	0.08797
XP_001624678	-5.47110	7.69795	8.05161	0.00455	0.08797
XP_003427871	5.70517	7.37240	8.03774	0.00458	0.08797
XP_003459722	5.70517	7.37240	8.03774	0.00458	0.08797
XP_796434	5.70517	7.37240	8.03774	0.00458	0.08797
AEK10750	-5.20312	9.99028	8.01498	0.00464	0.08797
XP_002607858	5.68708	7.35462	7.99585	0.00469	0.08797
ACN91277	-5.62891	7.01067	7.99144	0.00470	0.08797
ADX31291	-5.62891	7.01067	7.99144	0.00470	0.08797
BAE39125	-5.62891	7.01067	7.99144	0.00470	0.08797
CAI21694	-5.62891	7.01067	7.99144	0.00470	0.08797
XP_001121484	-5.62891	7.01067	7.99144	0.00470	0.08797
XP_001184164	-5.62891	7.01067	7.99144	0.00470	0.08797
XP_001188982	-5.62891	7.01067	7.99144	0.00470	0.08797
XP_001201290	-5.62891	7.01067	7.99144	0.00470	0.08797
XP_001863343	-5.62891	7.01067	7.99144	0.00470	0.08797
XP_002595371	-5.62891	7.01067	7.99144	0.00470	0.08797
XP_002610132	-5.62891	7.01067	7.99144	0.00470	0.08797

XP_003220431	-5.62891	7.01067	7.99144	0.00470	0.08797
XP_003223090	-5.62891	7.01067	7.99144	0.00470	0.08797
XP_003247452	-5.62891	7.01067	7.99144	0.00470	0.08797
XP_003293760	-5.62891	7.01067	7.99144	0.00470	0.08797
XP_793995	-5.62891	7.01067	7.99144	0.00470	0.08797
XP_001639778	5.66877	7.33662	7.95346	0.00480	0.08797
XP_002610097	5.66877	7.33662	7.95346	0.00480	0.08797
ABS88697	-5.59990	6.98180	7.92386	0.00488	0.08797
ACO08993	-5.59990	6.98180	7.92386	0.00488	0.08797
AEO36020	-5.59990	6.98180	7.92386	0.00488	0.08797
EFX73186	-5.59990	6.98180	7.92386	0.00488	0.08797
EFX88195	-5.59990	6.98180	7.92386	0.00488	0.08797
NP_001082284	-5.59990	6.98180	7.92386	0.00488	0.08797
NP_001128702	-5.59990	6.98180	7.92386	0.00488	0.08797
Q964E3	-5.59990	6.98180	7.92386	0.00488	0.08797
XP_001917051	-5.59990	6.98180	7.92386	0.00488	0.08797
XP_002431014	-5.59990	6.98180	7.92386	0.00488	0.08797
XP_002431121	-5.59990	6.98180	7.92386	0.00488	0.08797
XP_002606187	-5.59990	6.98180	7.92386	0.00488	0.08797
XP_002733477	-5.59990	6.98180	7.92386	0.00488	0.08797
XP_002733718	-5.59990	6.98180	7.92386	0.00488	0.08797
XP_002736186	-5.59990	6.98180	7.92386	0.00488	0.08797
XP_002741112	-5.59990	6.98180	7.92386	0.00488	0.08797
XP_003143113	-5.59990	6.98180	7.92386	0.00488	0.08797
XP_003397232	-5.59990	6.98180	7.92386	0.00488	0.08797
XP_003745138	-5.59990	6.98180	7.92386	0.00488	0.08797
XP_850799	-5.59990	6.98180	7.92386	0.00488	0.08797
YP_747305	-5.59990	6.98180	7.92386	0.00488	0.08797
AAH65041	5.65022	7.31839	7.91057	0.00491	0.08797
AAX27930	5.65022	7.31839	7.91057	0.00491	0.08797
AES05754	5.65022	7.31839	7.91057	0.00491	0.08797
BAA94854	5.65022	7.31839	7.91057	0.00491	0.08797
XP_002148768	5.65022	7.31839	7.91057	0.00491	0.08797
YP_002841142	5.65022	7.31839	7.91057	0.00491	0.08797
BAD99026	5.63143	7.29993	7.86717	0.00503	0.08797
NP_001139049	5.63143	7.29993	7.86717	0.00503	0.08797
XP_001313629	5.63143	7.29993	7.86717	0.00503	0.08797
XP_002590332	5.63143	7.29993	7.86717	0.00503	0.08797
XP_002680168	5.63143	7.29993	7.86717	0.00503	0.08797
XP_003443882	5.63143	7.29993	7.86717	0.00503	0.08797
3R2B_A	-5.57028	6.95235	7.85499	0.00507	0.08797
AAI61792	-5.57028	6.95235	7.85499	0.00507	0.08797
ACN91297	-5.57028	6.95235	7.85499	0.00507	0.08797
ADD18698	-5.57028	6.95235	7.85499	0.00507	0.08797

CAF33263	-5.57028	6.95235	7.85499	0.00507	0.08797
NP_001009986	-5.57028	6.95235	7.85499	0.00507	0.08797
XP_001636613	-5.57028	6.95235	7.85499	0.00507	0.08797
XP_001640172	-5.57028	6.95235	7.85499	0.00507	0.08797
XP_001647647	-5.57028	6.95235	7.85499	0.00507	0.08797
XP_001895717	-5.57028	6.95235	7.85499	0.00507	0.08797
XP_002131970	-5.57028	6.95235	7.85499	0.00507	0.08797
XP_002591357	-5.57028	6.95235	7.85499	0.00507	0.08797
XP_002603898	-5.57028	6.95235	7.85499	0.00507	0.08797
XP_002732174	-5.57028	6.95235	7.85499	0.00507	0.08797
XP_312429	-5.57028	6.95235	7.85499	0.00507	0.08797
XP_320464	-5.57028	6.95235	7.85499	0.00507	0.08797
XP_636792	-5.57028	6.95235	7.85499	0.00507	0.08797
XP_797423	-5.57028	6.95235	7.85499	0.00507	0.08797
EDL16829	5.61239	7.28123	7.82323	0.00516	0.08797
XP_002737958	5.61239	7.28123	7.82323	0.00516	0.08797
XP_003365356	5.61239	7.28123	7.82323	0.00516	0.08797
XP_629009	5.61239	7.28123	7.82323	0.00516	0.08797
XP_001019230	-5.54005	6.92228	7.78478	0.00527	0.08797
XP_001296210	-5.54005	6.92228	7.78478	0.00527	0.08797
XP_001329169	-5.54005	6.92228	7.78478	0.00527	0.08797
XP_002435466	-5.54005	6.92228	7.78478	0.00527	0.08797
XP_002593187	-5.54005	6.92228	7.78478	0.00527	0.08797
XP_002735710	-5.54005	6.92228	7.78478	0.00527	0.08797
XP_002736550	-5.54005	6.92228	7.78478	0.00527	0.08797
XP_002738667	-5.54005	6.92228	7.78478	0.00527	0.08797
XP_002739921	-5.54005	6.92228	7.78478	0.00527	0.08797
XP_003213845	-5.54005	6.92228	7.78478	0.00527	0.08797
XP_003498564	-5.54005	6.92228	7.78478	0.00527	0.08797
XP_424694	-5.54005	6.92228	7.78478	0.00527	0.08797
XP_002606696	5.59310	7.26229	7.77875	0.00529	0.08797
XP_003198228	5.59310	7.26229	7.77875	0.00529	0.08797
AAP41214	5.57354	7.24309	7.73371	0.00542	0.08797
XP_001316844	5.57354	7.24309	7.73371	0.00542	0.08797
XP_002730929	5.57354	7.24309	7.73371	0.00542	0.08797
XP_002912748	5.57354	7.24309	7.73371	0.00542	0.08797
CAI15956	-5.50917	6.89158	7.71317	0.00548	0.08797
DAA16422	-5.50917	6.89158	7.71317	0.00548	0.08797
EGW05886	-5.50917	6.89158	7.71317	0.00548	0.08797
EHA98399	-5.50917	6.89158	7.71317	0.00548	0.08797
EHB09998	-5.50917	6.89158	7.71317	0.00548	0.08797
XP_001351550	-5.50917	6.89158	7.71317	0.00548	0.08797
XP_001367489	-5.50917	6.89158	7.71317	0.00548	0.08797
XP_001629536	-5.50917	6.89158	7.71317	0.00548	0.08797

XP_001772366	-5.50917	6.89158	7.71317	0.00548	0.08797
XP_001842282	-5.50917	6.89158	7.71317	0.00548	0.08797
XP_001844033	-5.50917	6.89158	7.71317	0.00548	0.08797
XP_001994475	-5.50917	6.89158	7.71317	0.00548	0.08797
XP_002588882	-5.50917	6.89158	7.71317	0.00548	0.08797
XP_002592263	-5.50917	6.89158	7.71317	0.00548	0.08797
XP_002599089	-5.50917	6.89158	7.71317	0.00548	0.08797
XP_002605575	-5.50917	6.89158	7.71317	0.00548	0.08797
XP_002730499	-5.50917	6.89158	7.71317	0.00548	0.08797
XP_002739059	-5.50917	6.89158	7.71317	0.00548	0.08797
XP_002741281	-5.50917	6.89158	7.71317	0.00548	0.08797
XP_002742061	-5.50917	6.89158	7.71317	0.00548	0.08797
XP_002940872	-5.50917	6.89158	7.71317	0.00548	0.08797
XP_003731438	-5.50917	6.89158	7.71317	0.00548	0.08797
XP_646649	-5.50917	6.89158	7.71317	0.00548	0.08797
XP_788604	-5.50917	6.89158	7.71317	0.00548	0.08797
ZP_08205869	-5.50917	6.89158	7.71317	0.00548	0.08797
AAC00207	5.55372	7.22363	7.68810	0.00556	0.08797
AAI46846	5.55372	7.22363	7.68810	0.00556	0.08797
EFZ11915	5.55372	7.22363	7.68810	0.00556	0.08797
NP_001087022	5.55372	7.22363	7.68810	0.00556	0.08797
XP_002129966	5.55372	7.22363	7.68810	0.00556	0.08797
XP_002602847	5.55372	7.22363	7.68810	0.00556	0.08797
XP_002738789	5.55372	7.22363	7.68810	0.00556	0.08797
XP_003742500	5.55372	7.22363	7.68810	0.00556	0.08797
XP_973407	5.55372	7.22363	7.68810	0.00556	0.08797
YP_001958455	5.33893	7.87769	7.67378	0.00560	0.08797
AEO35323	5.53362	7.20391	7.64190	0.00570	0.08797
XP_002602373	5.53362	7.20391	7.64190	0.00570	0.08797
XP_002734510	5.53362	7.20391	7.64190	0.00570	0.08797
XP_002734543	5.53362	7.20391	7.64190	0.00570	0.08797
XP_002734661	5.53362	7.20391	7.64190	0.00570	0.08797
XP_002737120	5.53362	7.20391	7.64190	0.00570	0.08797
XP_003117370	5.53362	7.20391	7.64190	0.00570	0.08797
XP_003724058	5.53362	7.20391	7.64190	0.00570	0.08797
ADP08789	-5.47762	6.86021	7.64011	0.00571	0.08797
NP_001008648	-5.47762	6.86021	7.64011	0.00571	0.08797
NP_573311	-5.47762	6.86021	7.64011	0.00571	0.08797
XP_001375787	-5.47762	6.86021	7.64011	0.00571	0.08797
XP_001631865	-5.47762	6.86021	7.64011	0.00571	0.08797
XP_002115969	-5.47762	6.86021	7.64011	0.00571	0.08797
XP_002400122	-5.47762	6.86021	7.64011	0.00571	0.08797
XP_002596547	-5.47762	6.86021	7.64011	0.00571	0.08797
XP_002732306	-5.47762	6.86021	7.64011	0.00571	0.08797

XP_002735833	-5.47762	6.86021	7.64011	0.00571	0.08797
XP_002740846	-5.47762	6.86021	7.64011	0.00571	0.08797
XP_003729354	-5.47762	6.86021	7.64011	0.00571	0.08797
XP_783928	-5.47762	6.86021	7.64011	0.00571	0.08797
XP_787226	-5.47762	6.86021	7.64011	0.00571	0.08797
XP_970879	-5.47762	6.86021	7.64011	0.00571	0.08797
XP_002414754	-5.27902	7.50853	7.60378	0.00582	0.08797
1N0R_A	5.51324	7.18391	7.59510	0.00585	0.08797
AAF75839	5.51324	7.18391	7.59510	0.00585	0.08797
XP_002577332	5.51324	7.18391	7.59510	0.00585	0.08797
XP_002943235	5.51324	7.18391	7.59510	0.00585	0.08797
XP_395916	5.51324	7.18391	7.59510	0.00585	0.08797
AAH07747	-5.44536	6.82814	7.56554	0.00595	0.08797
ABF21059	-5.44536	6.82814	7.56554	0.00595	0.08797
AEO35704	-5.44536	6.82814	7.56554	0.00595	0.08797
CAB38180	-5.44536	6.82814	7.56554	0.00595	0.08797
EFX88686	-5.44536	6.82814	7.56554	0.00595	0.08797
EGI62678	-5.44536	6.82814	7.56554	0.00595	0.08797
EHB15871	-5.44536	6.82814	7.56554	0.00595	0.08797
NP_001072224	-5.44536	6.82814	7.56554	0.00595	0.08797
NP_001171814	-5.44536	6.82814	7.56554	0.00595	0.08797
XP_001013132	-5.44536	6.82814	7.56554	0.00595	0.08797
XP_001663322	-5.44536	6.82814	7.56554	0.00595	0.08797
XP_001914755	-5.44536	6.82814	7.56554	0.00595	0.08797
XP_002078082	-5.44536	6.82814	7.56554	0.00595	0.08797
XP_002427810	-5.44536	6.82814	7.56554	0.00595	0.08797
XP_002431913	-5.44536	6.82814	7.56554	0.00595	0.08797
XP_002603723	-5.44536	6.82814	7.56554	0.00595	0.08797
XP_002609740	-5.44536	6.82814	7.56554	0.00595	0.08797
XP_002613805	-5.44536	6.82814	7.56554	0.00595	0.08797
XP_002614074	-5.44536	6.82814	7.56554	0.00595	0.08797
XP_002732563	-5.44536	6.82814	7.56554	0.00595	0.08797
XP_002734654	-5.44536	6.82814	7.56554	0.00595	0.08797
XP_002738314	-5.44536	6.82814	7.56554	0.00595	0.08797
XP_002739885	-5.44536	6.82814	7.56554	0.00595	0.08797
XP_002831832	-5.44536	6.82814	7.56554	0.00595	0.08797
XP_003389851	-5.44536	6.82814	7.56554	0.00595	0.08797
XP_003437908	-5.44536	6.82814	7.56554	0.00595	0.08797
XP_003490045	-5.44536	6.82814	7.56554	0.00595	0.08797
XP_003494875	-5.44536	6.82814	7.56554	0.00595	0.08797
XP_003727446	-5.44536	6.82814	7.56554	0.00595	0.08797
XP_417571	-5.44536	6.82814	7.56554	0.00595	0.08797
ZP_09965478	-5.44536	6.82814	7.56554	0.00595	0.08797
EFX86378	5.49256	7.16363	7.54769	0.00601	0.08806

XP_001379766	5.49256	7.16363	7.54769	0.00601	0.08806
XP_002736138	5.49256	7.16363	7.54769	0.00601	0.08806
XP_003445167	5.49256	7.16363	7.54769	0.00601	0.08806
XP_003731784	5.49256	7.16363	7.54769	0.00601	0.08806
XP_319948	5.49256	7.16363	7.54769	0.00601	0.08806
XP_675648	5.49256	7.16363	7.54769	0.00601	0.08806
XP_002731452	-5.23733	7.46747	7.50711	0.00615	0.08840
AAH42646	5.47159	7.14306	7.49964	0.00617	0.08840
XP_001629191	5.47159	7.14306	7.49964	0.00617	0.08840
XP_001657105	5.47159	7.14306	7.49964	0.00617	0.08840
XP_002732972	5.47159	7.14306	7.49964	0.00617	0.08840
XP_003216403	5.47159	7.14306	7.49964	0.00617	0.08840
XP_003503117	5.47159	7.14306	7.49964	0.00617	0.08840
AAH76415	-5.41236	6.79534	7.48938	0.00621	0.08840
BAE79743	-5.41236	6.79534	7.48938	0.00621	0.08840
EFZ21157	-5.41236	6.79534	7.48938	0.00621	0.08840
P86856	-5.41236	6.79534	7.48938	0.00621	0.08840
Q86MA7	-5.41236	6.79534	7.48938	0.00621	0.08840
XP_001622679	-5.41236	6.79534	7.48938	0.00621	0.08840
XP_001638741	-5.41236	6.79534	7.48938	0.00621	0.08840
XP_001639688	-5.41236	6.79534	7.48938	0.00621	0.08840
XP_002607271	-5.41236	6.79534	7.48938	0.00621	0.08840
XP_003730972	-5.41236	6.79534	7.48938	0.00621	0.08840
XP_003731756	-5.41236	6.79534	7.48938	0.00621	0.08840
XP_312357	-5.41236	6.79534	7.48938	0.00621	0.08840
XP_781600	-5.41236	6.79534	7.48938	0.00621	0.08840
XP_790269	-5.41236	6.79534	7.48938	0.00621	0.08840
XP_793314	-5.41236	6.79534	7.48938	0.00621	0.08840
YP_004894149	-5.41236	6.79534	7.48938	0.00621	0.08840
CAX13385	5.45030	7.12219	7.45094	0.00634	0.08911
CCD77454	5.45030	7.12219	7.45094	0.00634	0.08911
EFN63056	5.45030	7.12219	7.45094	0.00634	0.08911
NP_001158362	5.45030	7.12219	7.45094	0.00634	0.08911
XP_002603103	5.45030	7.12219	7.45094	0.00634	0.08911
XP_002608536	5.45030	7.12219	7.45094	0.00634	0.08911
XP_002751314	5.45030	7.12219	7.45094	0.00634	0.08911
XP_003240425	5.45030	7.12219	7.45094	0.00634	0.08911
XP_785648	5.45030	7.12219	7.45094	0.00634	0.08911
ZP_04532941	5.45030	7.12219	7.45094	0.00634	0.08911
ZP_08102074	5.45030	7.12219	7.45094	0.00634	0.08911
XP_002426989	-5.11568	7.87796	7.41962	0.00645	0.08935
BAB27697	-5.37858	6.76178	7.41158	0.00648	0.08935
BAH12781	-5.37858	6.76178	7.41158	0.00648	0.08935
BAJ24842	-5.37858	6.76178	7.41158	0.00648	0.08935

DAA34668	-5.37858	6.76178	7.41158	0.00648	0.08935
H2A0M0	-5.37858	6.76178	7.41158	0.00648	0.08935
NP_001121513	-5.37858	6.76178	7.41158	0.00648	0.08935
NP_724543	-5.37858	6.76178	7.41158	0.00648	0.08935
XP_001491293	-5.37858	6.76178	7.41158	0.00648	0.08935
XP_002416006	-5.37858	6.76178	7.41158	0.00648	0.08935
XP_002586063	-5.37858	6.76178	7.41158	0.00648	0.08935
XP_002605569	-5.37858	6.76178	7.41158	0.00648	0.08935
XP_002739909	-5.37858	6.76178	7.41158	0.00648	0.08935
XP_003724122	-5.37858	6.76178	7.41158	0.00648	0.08935
XP_796961	-5.37858	6.76178	7.41158	0.00648	0.08935
ABJ97377	5.42869	7.10102	7.40157	0.00652	0.08935
CAI11834	5.42869	7.10102	7.40157	0.00652	0.08935
NP_001128543	5.42869	7.10102	7.40157	0.00652	0.08935
XP_002604515	5.42869	7.10102	7.40157	0.00652	0.08935
XP_796030	5.42869	7.10102	7.40157	0.00652	0.08935
AFC98245	-5.05924	8.20967	7.40096	0.00652	0.08935
XP_002741846	5.40676	7.07953	7.35151	0.00670	0.09092
XP_003382559	5.40676	7.07953	7.35151	0.00670	0.09092
XP_003440100	5.40676	7.07953	7.35151	0.00670	0.09092
XP_003727967	5.40676	7.07953	7.35151	0.00670	0.09092
AAC46490	-5.34400	6.72743	7.33207	0.00677	0.09092
AEO34511	-5.34400	6.72743	7.33207	0.00677	0.09092
BAF63789	-5.34400	6.72743	7.33207	0.00677	0.09092
EGI70977	-5.34400	6.72743	7.33207	0.00677	0.09092
NP_001191639	-5.34400	6.72743	7.33207	0.00677	0.09092
XP_002130339	-5.34400	6.72743	7.33207	0.00677	0.09092
XP_002131378	-5.34400	6.72743	7.33207	0.00677	0.09092
XP_002601230	-5.34400	6.72743	7.33207	0.00677	0.09092
XP_002733462	-5.34400	6.72743	7.33207	0.00677	0.09092
XP_002738807	-5.34400	6.72743	7.33207	0.00677	0.09092
XP_002989592	-5.34400	6.72743	7.33207	0.00677	0.09092
XP_003244885	-5.34400	6.72743	7.33207	0.00677	0.09092
XP_414938	-5.34400	6.72743	7.33207	0.00677	0.09092
XP_800759	-5.34400	6.72743	7.33207	0.00677	0.09092
AAI61240	5.38449	7.05772	7.30075	0.00689	0.09177
BAH84829	5.38449	7.05772	7.30075	0.00689	0.09177
EFN81652	5.38449	7.05772	7.30075	0.00689	0.09177
XP_002128280	5.38449	7.05772	7.30075	0.00689	0.09177
XP_002319013	5.38449	7.05772	7.30075	0.00689	0.09177
XP_002427237	5.38449	7.05772	7.30075	0.00689	0.09177
XP_317010	5.38449	7.05772	7.30075	0.00689	0.09177
XP_002607353	4.94504	9.19652	7.27924	0.00698	0.09192
AAD12246	-5.30857	6.69223	7.25075	0.00709	0.09192

AAH08328	-5.30857	6.69223	7.25075	0.00709	0.09192
AFE74132	-5.30857	6.69223	7.25075	0.00709	0.09192
EFB18688	-5.30857	6.69223	7.25075	0.00709	0.09192
JC8022	-5.30857	6.69223	7.25075	0.00709	0.09192
NP_001139570	-5.30857	6.69223	7.25075	0.00709	0.09192
NP_001242994	-5.30857	6.69223	7.25075	0.00709	0.09192
XP_001363658	-5.30857	6.69223	7.25075	0.00709	0.09192
XP_002187407	-5.30857	6.69223	7.25075	0.00709	0.09192
XP_002595765	-5.30857	6.69223	7.25075	0.00709	0.09192
XP_002595800	-5.30857	6.69223	7.25075	0.00709	0.09192
XP_002718217	-5.30857	6.69223	7.25075	0.00709	0.09192
XP_002731212	-5.30857	6.69223	7.25075	0.00709	0.09192
XP_003453111	-5.30857	6.69223	7.25075	0.00709	0.09192
XP_787033	-5.30857	6.69223	7.25075	0.00709	0.09192
XP_795044	-5.30857	6.69223	7.25075	0.00709	0.09192
XP_968982	-5.30857	6.69223	7.25075	0.00709	0.09192
XP_969067	-5.30857	6.69223	7.25075	0.00709	0.09192
YP_629985	-5.30857	6.69223	7.25075	0.00709	0.09192
ZP_01453154	-5.30857	6.69223	7.25075	0.00709	0.09192
CAA69658	5.36187	7.03557	7.24926	0.00709	0.09192
XP_002938053	5.36187	7.03557	7.24926	0.00709	0.09192
XP_003345830	5.36187	7.03557	7.24926	0.00709	0.09192
AAV85465	4.97062	8.75321	7.24129	0.00712	0.09222
AAC35953	-4.94694	8.40500	7.21406	0.00723	0.09309
AAH81274	5.33889	7.01307	7.19702	0.00730	0.09309
XP_002578551	5.33889	7.01307	7.19702	0.00730	0.09309
XP_002589335	5.33889	7.01307	7.19702	0.00730	0.09309
XP_002590403	5.33889	7.01307	7.19702	0.00730	0.09309
XP_002607686	5.33889	7.01307	7.19702	0.00730	0.09309
XP_002919141	5.33889	7.01307	7.19702	0.00730	0.09309
XP_003494521	5.33889	7.01307	7.19702	0.00730	0.09309
XP_003723431	5.33889	7.01307	7.19702	0.00730	0.09309
XP_003725724	5.33889	7.01307	7.19702	0.00730	0.09309
ACU83221	-5.27225	6.65616	7.16756	0.00742	0.09309
AEG78368	-5.27225	6.65616	7.16756	0.00742	0.09309
AEO32292	-5.27225	6.65616	7.16756	0.00742	0.09309
BAH14572	-5.27225	6.65616	7.16756	0.00742	0.09309
CAC82191	-5.27225	6.65616	7.16756	0.00742	0.09309
EAW68433	-5.27225	6.65616	7.16756	0.00742	0.09309
EFX84014	-5.27225	6.65616	7.16756	0.00742	0.09309
EFZ15737	-5.27225	6.65616	7.16756	0.00742	0.09309
NP_001134210	-5.27225	6.65616	7.16756	0.00742	0.09309
XP_001627242	-5.27225	6.65616	7.16756	0.00742	0.09309
XP_002109086	-5.27225	6.65616	7.16756	0.00742	0.09309

XP_002131963	-5.27225	6.65616	7.16756	0.00742	0.09309
XP_002590806	-5.27225	6.65616	7.16756	0.00742	0.09309
XP_002735979	-5.27225	6.65616	7.16756	0.00742	0.09309
XP_002738454	-5.27225	6.65616	7.16756	0.00742	0.09309
XP_003729203	-5.27225	6.65616	7.16756	0.00742	0.09309
XP_003742214	-5.27225	6.65616	7.16756	0.00742	0.09309
XP_793154	-5.27225	6.65616	7.16756	0.00742	0.09309
ZP_09232839	-5.27225	6.65616	7.16756	0.00742	0.09309
XP_002609800	-5.08115	7.31380	7.14676	0.00751	0.09331
BAC34619	5.31554	6.99022	7.14402	0.00752	0.09331
XP_001021944	5.31554	6.99022	7.14402	0.00752	0.09331
XP_002196829	5.31554	6.99022	7.14402	0.00752	0.09331
XP_002595898	5.31554	6.99022	7.14402	0.00752	0.09331
XP_002738406	5.31554	6.99022	7.14402	0.00752	0.09331
XP_002741442	5.31554	6.99022	7.14402	0.00752	0.09331
XP_003515270	5.31554	6.99022	7.14402	0.00752	0.09331
XP_003726678	5.31554	6.99022	7.14402	0.00752	0.09331
XP_396524	5.31554	6.99022	7.14402	0.00752	0.09331
AAI05210	5.29180	6.96700	7.09022	0.00775	0.09358
EDL09901	5.29180	6.96700	7.09022	0.00775	0.09358
EHJ78503	5.29180	6.96700	7.09022	0.00775	0.09358
XP_002733315	5.29180	6.96700	7.09022	0.00775	0.09358
XP_002737235	5.29180	6.96700	7.09022	0.00775	0.09358
XP_002741523	5.29180	6.96700	7.09022	0.00775	0.09358
XP_003219674	5.29180	6.96700	7.09022	0.00775	0.09358
XP_003440255	5.29180	6.96700	7.09022	0.00775	0.09358
XP_623578	5.29180	6.96700	7.09022	0.00775	0.09358
XP_625002	5.29180	6.96700	7.09022	0.00775	0.09358
ADQ43243	-5.23499	6.61917	7.08240	0.00778	0.09358
BAH70506	-5.23499	6.61917	7.08240	0.00778	0.09358
BAM17961	-5.23499	6.61917	7.08240	0.00778	0.09358
C3YWU0	-5.23499	6.61917	7.08240	0.00778	0.09358
CAJ38815	-5.23499	6.61917	7.08240	0.00778	0.09358
EFX84334	-5.23499	6.61917	7.08240	0.00778	0.09358
NP_001079840	-5.23499	6.61917	7.08240	0.00778	0.09358
XP_002081583	-5.23499	6.61917	7.08240	0.00778	0.09358
XP_002167634	-5.23499	6.61917	7.08240	0.00778	0.09358
XP_002410016	-5.23499	6.61917	7.08240	0.00778	0.09358
XP_002611888	-5.23499	6.61917	7.08240	0.00778	0.09358
XP_002716385	-5.23499	6.61917	7.08240	0.00778	0.09358
XP_002732810	-5.23499	6.61917	7.08240	0.00778	0.09358
XP_002741428	-5.23499	6.61917	7.08240	0.00778	0.09358
XP_002904935	-5.23499	6.61917	7.08240	0.00778	0.09358
XP_003284143	-5.23499	6.61917	7.08240	0.00778	0.09358

XP_003447492	-5.23499	6.61917	7.08240	0.00778	0.09358
XP_003455212	-5.23499	6.61917	7.08240	0.00778	0.09358
XP_644124	-5.23499	6.61917	7.08240	0.00778	0.09358
XP_728306	-5.23499	6.61917	7.08240	0.00778	0.09358
AAH23438	5.26766	6.94340	7.03560	0.00799	0.09459
CAD79439	5.26766	6.94340	7.03560	0.00799	0.09459
NP_001028276	5.26766	6.94340	7.03560	0.00799	0.09459
XP_002121377	5.26766	6.94340	7.03560	0.00799	0.09459
XP_002603744	5.26766	6.94340	7.03560	0.00799	0.09459
XP_002922184	5.26766	6.94340	7.03560	0.00799	0.09459
XP_003704775	5.26766	6.94340	7.03560	0.00799	0.09459
ACO09839	-5.19674	6.58120	6.99518	0.00817	0.09459
ADI78068	-5.19674	6.58120	6.99518	0.00817	0.09459
AES07133	-5.19674	6.58120	6.99518	0.00817	0.09459
BAK52806	-5.19674	6.58120	6.99518	0.00817	0.09459
BAK61936	-5.19674	6.58120	6.99518	0.00817	0.09459
BAM20344	-5.19674	6.58120	6.99518	0.00817	0.09459
EFN86147	-5.19674	6.58120	6.99518	0.00817	0.09459
EGT46744	-5.19674	6.58120	6.99518	0.00817	0.09459
EGU89445	-5.19674	6.58120	6.99518	0.00817	0.09459
NP_001120175	-5.19674	6.58120	6.99518	0.00817	0.09459
NP_001161644	-5.19674	6.58120	6.99518	0.00817	0.09459
NP_001187438	-5.19674	6.58120	6.99518	0.00817	0.09459
XP_001634032	-5.19674	6.58120	6.99518	0.00817	0.09459
XP_002596805	-5.19674	6.58120	6.99518	0.00817	0.09459
XP_002605354	-5.19674	6.58120	6.99518	0.00817	0.09459
XP_002609809	-5.19674	6.58120	6.99518	0.00817	0.09459
XP_002610578	-5.19674	6.58120	6.99518	0.00817	0.09459
XP_002611948	-5.19674	6.58120	6.99518	0.00817	0.09459
XP_002736837	-5.19674	6.58120	6.99518	0.00817	0.09459
XP_002737332	-5.19674	6.58120	6.99518	0.00817	0.09459
XP_002742014	-5.19674	6.58120	6.99518	0.00817	0.09459
XP_002918754	-5.19674	6.58120	6.99518	0.00817	0.09459
XP_003383371	-5.19674	6.58120	6.99518	0.00817	0.09459
XP_003706967	-5.19674	6.58120	6.99518	0.00817	0.09459
XP_003728329	-5.19674	6.58120	6.99518	0.00817	0.09459
XP_652421	-5.19674	6.58120	6.99518	0.00817	0.09459
XP_788973	-5.19674	6.58120	6.99518	0.00817	0.09459
XP_797784	-5.19674	6.58120	6.99518	0.00817	0.09459
AAF08010	5.24312	6.91940	6.98014	0.00824	0.09459
AAI30064	5.24312	6.91940	6.98014	0.00824	0.09459
ABN58714	5.24312	6.91940	6.98014	0.00824	0.09459
EHB18107	5.24312	6.91940	6.98014	0.00824	0.09459
NP_001096464	5.24312	6.91940	6.98014	0.00824	0.09459

XP_001630804	5.24312	6.91940	6.98014	0.00824	0.09459
XP_001638183	5.24312	6.91940	6.98014	0.00824	0.09459
XP_002427605	5.24312	6.91940	6.98014	0.00824	0.09459
XP_002732982	5.24312	6.91940	6.98014	0.00824	0.09459
XP_002741356	5.24312	6.91940	6.98014	0.00824	0.09459
XP_003706024	5.24312	6.91940	6.98014	0.00824	0.09459
EFN77118	-4.90977	7.67650	6.94526	0.00840	0.09504
AEF33431	5.21815	6.89500	6.92381	0.00851	0.09504
EGZ11335	5.21815	6.89500	6.92381	0.00851	0.09504
XP_002169156	5.21815	6.89500	6.92381	0.00851	0.09504
XP_002423593	5.21815	6.89500	6.92381	0.00851	0.09504
XP_002427334	5.21815	6.89500	6.92381	0.00851	0.09504
XP_002603725	5.21815	6.89500	6.92381	0.00851	0.09504
XP_002609157	5.21815	6.89500	6.92381	0.00851	0.09504
XP_002612048	5.21815	6.89500	6.92381	0.00851	0.09504
XP_002737647	5.21815	6.89500	6.92381	0.00851	0.09504
XP_003199942	5.21815	6.89500	6.92381	0.00851	0.09504
XP_003201532	5.21815	6.89500	6.92381	0.00851	0.09504
XP_003451110	5.21815	6.89500	6.92381	0.00851	0.09504
AAB94002	-5.15745	6.54221	6.90580	0.00859	0.09504
AAX59986	-5.15745	6.54221	6.90580	0.00859	0.09504
CAA03852	-5.15745	6.54221	6.90580	0.00859	0.09504
CAH72156	-5.15745	6.54221	6.90580	0.00859	0.09504
CAM16369	-5.15745	6.54221	6.90580	0.00859	0.09504
CCC90229	-5.15745	6.54221	6.90580	0.00859	0.09504
EAW92073	-5.15745	6.54221	6.90580	0.00859	0.09504
EHB01690	-5.15745	6.54221	6.90580	0.00859	0.09504
XP_001073612	-5.15745	6.54221	6.90580	0.00859	0.09504
XP_001370030	-5.15745	6.54221	6.90580	0.00859	0.09504
XP_001622790	-5.15745	6.54221	6.90580	0.00859	0.09504
XP_001730439	-5.15745	6.54221	6.90580	0.00859	0.09504
XP_002121304	-5.15745	6.54221	6.90580	0.00859	0.09504
XP_002594115	-5.15745	6.54221	6.90580	0.00859	0.09504
XP_002594726	-5.15745	6.54221	6.90580	0.00859	0.09504
XP_002599337	-5.15745	6.54221	6.90580	0.00859	0.09504
XP_002606340	-5.15745	6.54221	6.90580	0.00859	0.09504
XP_002606484	-5.15745	6.54221	6.90580	0.00859	0.09504
XP_002606952	-5.15745	6.54221	6.90580	0.00859	0.09504
XP_002607276	-5.15745	6.54221	6.90580	0.00859	0.09504
XP_002613830	-5.15745	6.54221	6.90580	0.00859	0.09504
XP_002737170	-5.15745	6.54221	6.90580	0.00859	0.09504
XP_002740120	-5.15745	6.54221	6.90580	0.00859	0.09504
XP_003459606	-5.15745	6.54221	6.90580	0.00859	0.09504
XP_003743136	-5.15745	6.54221	6.90580	0.00859	0.09504

AAM12624	5.19274	6.87018	6.86659	0.00878	0.09607
ABF18435	5.19274	6.87018	6.86659	0.00878	0.09607
BAI78310	5.19274	6.87018	6.86659	0.00878	0.09607
XP_001655365	5.19274	6.87018	6.86659	0.00878	0.09607
XP_001917505	5.19274	6.87018	6.86659	0.00878	0.09607
XP_002597495	5.19274	6.87018	6.86659	0.00878	0.09607
XP_002603507	5.19274	6.87018	6.86659	0.00878	0.09607
XP_700884	5.19274	6.87018	6.86659	0.00878	0.09607
XP_002735254	-4.82453	7.98076	6.86034	0.00881	0.09607
XP_003704588	-4.82453	7.98076	6.86034	0.00881	0.09607
AAN71247	-5.11706	6.50214	6.81414	0.00904	0.09607
AAO16597	-5.11706	6.50214	6.81414	0.00904	0.09607
ABJ17046	-5.11706	6.50214	6.81414	0.00904	0.09607
ABZ04225	-5.11706	6.50214	6.81414	0.00904	0.09607
ACN25141	-5.11706	6.50214	6.81414	0.00904	0.09607
ACY92475	-5.11706	6.50214	6.81414	0.00904	0.09607
ADY43920	-5.11706	6.50214	6.81414	0.00904	0.09607
EFN60550	-5.11706	6.50214	6.81414	0.00904	0.09607
EIM88312	-5.11706	6.50214	6.81414	0.00904	0.09607
XP_001623400	-5.11706	6.50214	6.81414	0.00904	0.09607
XP_001624183	-5.11706	6.50214	6.81414	0.00904	0.09607
XP_002191916	-5.11706	6.50214	6.81414	0.00904	0.09607
XP_002599532	-5.11706	6.50214	6.81414	0.00904	0.09607
XP_002603910	-5.11706	6.50214	6.81414	0.00904	0.09607
XP_002613713	-5.11706	6.50214	6.81414	0.00904	0.09607
XP_002730816	-5.11706	6.50214	6.81414	0.00904	0.09607
XP_002735108	-5.11706	6.50214	6.81414	0.00904	0.09607
XP_002737296	-5.11706	6.50214	6.81414	0.00904	0.09607
XP_002739880	-5.11706	6.50214	6.81414	0.00904	0.09607
XP_002740327	-5.11706	6.50214	6.81414	0.00904	0.09607
XP_002941352	-5.11706	6.50214	6.81414	0.00904	0.09607
XP_003230829	-5.11706	6.50214	6.81414	0.00904	0.09607
XP_003249185	-5.11706	6.50214	6.81414	0.00904	0.09607
XP_003377489	-5.11706	6.50214	6.81414	0.00904	0.09607
XP_003416137	-5.11706	6.50214	6.81414	0.00904	0.09607
XP_792701	-5.11706	6.50214	6.81414	0.00904	0.09607
ABK63640	5.16687	6.84493	6.80844	0.00907	0.09607
AFA34361	5.16687	6.84493	6.80844	0.00907	0.09607
CAF96009	5.16687	6.84493	6.80844	0.00907	0.09607
NP_001177233	5.16687	6.84493	6.80844	0.00907	0.09607
XP_001638576	5.16687	6.84493	6.80844	0.00907	0.09607
XP_002118044	5.16687	6.84493	6.80844	0.00907	0.09607
XP_002120553	5.16687	6.84493	6.80844	0.00907	0.09607
XP_002419737	5.16687	6.84493	6.80844	0.00907	0.09607

XP_002731635	5.16687	6.84493	6.80844	0.00907	0.09607
XP_003412872	5.16687	6.84493	6.80844	0.00907	0.09607
XP_003443814	5.16687	6.84493	6.80844	0.00907	0.09607
BAE36201	5.14054	6.81922	6.74934	0.00938	0.09740
CAQ14975	5.14054	6.81922	6.74934	0.00938	0.09740
NP_001011952	5.14054	6.81922	6.74934	0.00938	0.09740
XP_002524490	5.14054	6.81922	6.74934	0.00938	0.09740
XP_002586461	5.14054	6.81922	6.74934	0.00938	0.09740
XP_002597866	5.14054	6.81922	6.74934	0.00938	0.09740
XP_002740084	5.14054	6.81922	6.74934	0.00938	0.09740
XP_003514888	5.14054	6.81922	6.74934	0.00938	0.09740
AAH20198	-5.07551	6.46092	6.72009	0.00953	0.09740
AAI42797	-5.07551	6.46092	6.72009	0.00953	0.09740
AAX26856	-5.07551	6.46092	6.72009	0.00953	0.09740
AES11373	-5.07551	6.46092	6.72009	0.00953	0.09740
BAE00688	-5.07551	6.46092	6.72009	0.00953	0.09740
CAG05170	-5.07551	6.46092	6.72009	0.00953	0.09740
CBX41740	-5.07551	6.46092	6.72009	0.00953	0.09740
DAA01766	-5.07551	6.46092	6.72009	0.00953	0.09740
EFX90172	-5.07551	6.46092	6.72009	0.00953	0.09740
P25324	-5.07551	6.46092	6.72009	0.00953	0.09740
XP_001630985	-5.07551	6.46092	6.72009	0.00953	0.09740
XP_001632595	-5.07551	6.46092	6.72009	0.00953	0.09740
XP_001648420	-5.07551	6.46092	6.72009	0.00953	0.09740
XP_001650130	-5.07551	6.46092	6.72009	0.00953	0.09740
XP_001747261	-5.07551	6.46092	6.72009	0.00953	0.09740
XP_001811118	-5.07551	6.46092	6.72009	0.00953	0.09740
XP_001901240	-5.07551	6.46092	6.72009	0.00953	0.09740
XP_002107929	-5.07551	6.46092	6.72009	0.00953	0.09740
XP_002589568	-5.07551	6.46092	6.72009	0.00953	0.09740
XP_002595454	-5.07551	6.46092	6.72009	0.00953	0.09740
XP_002599181	-5.07551	6.46092	6.72009	0.00953	0.09740
XP_002602987	-5.07551	6.46092	6.72009	0.00953	0.09740
XP_002608254	-5.07551	6.46092	6.72009	0.00953	0.09740
XP_002671735	-5.07551	6.46092	6.72009	0.00953	0.09740
XP_002708625	-5.07551	6.46092	6.72009	0.00953	0.09740
XP_002732024	-5.07551	6.46092	6.72009	0.00953	0.09740
XP_002734349	-5.07551	6.46092	6.72009	0.00953	0.09740
XP_003224930	-5.07551	6.46092	6.72009	0.00953	0.09740
XP_003486382	-5.07551	6.46092	6.72009	0.00953	0.09740
XP_003693496	-5.07551	6.46092	6.72009	0.00953	0.09740
XP_392313	-5.07551	6.46092	6.72009	0.00953	0.09740
YP_501570	-5.07551	6.46092	6.72009	0.00953	0.09740
XP_001861636	-4.66810	8.95223	6.71439	0.00956	0.09763

AAH56009	5.11371	6.79305	6.68925	0.00970	0.09798
AAS86702	5.11371	6.79305	6.68925	0.00970	0.09798
ABR68007	5.11371	6.79305	6.68925	0.00970	0.09798
ACL68661	5.11371	6.79305	6.68925	0.00970	0.09798
ADU19852	5.11371	6.79305	6.68925	0.00970	0.09798
AEF33398	5.11371	6.79305	6.68925	0.00970	0.09798
AEO33549	5.11371	6.79305	6.68925	0.00970	0.09798
CAX14245	5.11371	6.79305	6.68925	0.00970	0.09798
XP_001098430	5.11371	6.79305	6.68925	0.00970	0.09798
XP_002190622	5.11371	6.79305	6.68925	0.00970	0.09798
XP_002585735	5.11371	6.79305	6.68925	0.00970	0.09798
XP_002741525	5.11371	6.79305	6.68925	0.00970	0.09798
AAL40415	-4.59598	10.01389	6.65657	0.00988	0.09868
3ZU7_B	5.08637	6.76639	6.62814	0.01004	0.09868
ACA53359	5.08637	6.76639	6.62814	0.01004	0.09868
AEO34496	5.08637	6.76639	6.62814	0.01004	0.09868
BAB24599	5.08637	6.76639	6.62814	0.01004	0.09868
NP_001003525	5.08637	6.76639	6.62814	0.01004	0.09868
NP_001246171	5.08637	6.76639	6.62814	0.01004	0.09868
XP_002733177	5.08637	6.76639	6.62814	0.01004	0.09868
XP_002735577	5.08637	6.76639	6.62814	0.01004	0.09868
XP_003218952	5.08637	6.76639	6.62814	0.01004	0.09868
XP_003444610	5.08637	6.76639	6.62814	0.01004	0.09868
CAF95840	-5.03272	6.41850	6.62353	0.01006	0.09868
DAA01286	-5.03272	6.41850	6.62353	0.01006	0.09868
EFR22584	-5.03272	6.41850	6.62353	0.01006	0.09868
NP_001073476	-5.03272	6.41850	6.62353	0.01006	0.09868
NP_997822	-5.03272	6.41850	6.62353	0.01006	0.09868
XP_001381503	-5.03272	6.41850	6.62353	0.01006	0.09868
XP_001600405	-5.03272	6.41850	6.62353	0.01006	0.09868
XP_001623090	-5.03272	6.41850	6.62353	0.01006	0.09868
XP_002161139	-5.03272	6.41850	6.62353	0.01006	0.09868
XP_002422774	-5.03272	6.41850	6.62353	0.01006	0.09868
XP_002431934	-5.03272	6.41850	6.62353	0.01006	0.09868
XP_002593165	-5.03272	6.41850	6.62353	0.01006	0.09868
XP_002612124	-5.03272	6.41850	6.62353	0.01006	0.09868
XP_002730573	-5.03272	6.41850	6.62353	0.01006	0.09868
XP_002735319	-5.03272	6.41850	6.62353	0.01006	0.09868
XP_002742026	-5.03272	6.41850	6.62353	0.01006	0.09868
XP_003215978	-5.03272	6.41850	6.62353	0.01006	0.09868
XP_003227281	-5.03272	6.41850	6.62353	0.01006	0.09868
XP_003228880	-5.03272	6.41850	6.62353	0.01006	0.09868
XP_003437657	-5.03272	6.41850	6.62353	0.01006	0.09868
XP_003480569	-5.03272	6.41850	6.62353	0.01006	0.09868

XP_623653	-5.03272	6.41850	6.62353	0.01006	0.09868
XP_968186	-5.03272	6.41850	6.62353	0.01006	0.09868
XP_001509166	-4.85175	7.08866	6.62322	0.01007	0.09868
AAH48199	5.05851	6.73923	6.56597	0.01039	0.09978
EDL08506	5.05851	6.73923	6.56597	0.01039	0.09978
EFN87015	5.05851	6.73923	6.56597	0.01039	0.09978
EFX89628	5.05851	6.73923	6.56597	0.01039	0.09978
EHJ77866	5.05851	6.73923	6.56597	0.01039	0.09978
NP_001085695	5.05851	6.73923	6.56597	0.01039	0.09978
NP_001087707	5.05851	6.73923	6.56597	0.01039	0.09978
NP_758033	5.05851	6.73923	6.56597	0.01039	0.09978
XP_001371296	5.05851	6.73923	6.56597	0.01039	0.09978
XP_002076869	5.05851	6.73923	6.56597	0.01039	0.09978
XP_002586024	5.05851	6.73923	6.56597	0.01039	0.09978
XP_002592162	5.05851	6.73923	6.56597	0.01039	0.09978
XP_002592437	5.05851	6.73923	6.56597	0.01039	0.09978
XP_002600896	5.05851	6.73923	6.56597	0.01039	0.09978
XP_002607406	5.05851	6.73923	6.56597	0.01039	0.09978
XP_003443102	5.05851	6.73923	6.56597	0.01039	0.09978
XP_003726012	5.05851	6.73923	6.56597	0.01039	0.09978
XP_003746101	5.05851	6.73923	6.56597	0.01039	0.09978
XP_782883	5.05851	6.73923	6.56597	0.01039	0.09978
XP_787047	5.05851	6.73923	6.56597	0.01039	0.09978
XP_003389557	-4.82386	7.06133	6.56007	0.01043	0.09978
XP_003382892	-4.62174	8.55560	6.55695	0.01045	0.09978
AAG42824	-4.98863	6.37479	6.52430	0.01064	0.09978
AAI42789	-4.98863	6.37479	6.52430	0.01064	0.09978
AAK96227	-4.98863	6.37479	6.52430	0.01064	0.09978
AAM18869	-4.98863	6.37479	6.52430	0.01064	0.09978
AAT97079	-4.98863	6.37479	6.52430	0.01064	0.09978
AAW24718	-4.98863	6.37479	6.52430	0.01064	0.09978
AEO34520	-4.98863	6.37479	6.52430	0.01064	0.09978
CAP19429	-4.98863	6.37479	6.52430	0.01064	0.09978
EAW89598	-4.98863	6.37479	6.52430	0.01064	0.09978
EGD80777	-4.98863	6.37479	6.52430	0.01064	0.09978
NP_001103594	-4.98863	6.37479	6.52430	0.01064	0.09978
NP_001177260	-4.98863	6.37479	6.52430	0.01064	0.09978
XP_001601734	-4.98863	6.37479	6.52430	0.01064	0.09978
XP_001635714	-4.98863	6.37479	6.52430	0.01064	0.09978
XP_001640621	-4.98863	6.37479	6.52430	0.01064	0.09978
XP_001659775	-4.98863	6.37479	6.52430	0.01064	0.09978
XP_001956625	-4.98863	6.37479	6.52430	0.01064	0.09978
XP_002105029	-4.98863	6.37479	6.52430	0.01064	0.09978
XP_002588214	-4.98863	6.37479	6.52430	0.01064	0.09978

XP_002589790	-4.98863	6.37479	6.52430	0.01064	0.09978
XP_002593069	-4.98863	6.37479	6.52430	0.01064	0.09978
XP_002601232	-4.98863	6.37479	6.52430	0.01064	0.09978
XP_002601620	-4.98863	6.37479	6.52430	0.01064	0.09978
XP_002732898	-4.98863	6.37479	6.52430	0.01064	0.09978
XP_002738217	-4.98863	6.37479	6.52430	0.01064	0.09978
XP_002834412	-4.98863	6.37479	6.52430	0.01064	0.09978
XP_003230802	-4.98863	6.37479	6.52430	0.01064	0.09978
XP_003243638	-4.98863	6.37479	6.52430	0.01064	0.09978
XP_003384634	-4.98863	6.37479	6.52430	0.01064	0.09978
XP_003726964	-4.98863	6.37479	6.52430	0.01064	0.09978
XP_785073	-4.98863	6.37479	6.52430	0.01064	0.09978
XP_790246	-4.98863	6.37479	6.52430	0.01064	0.09978
CCD78935	5.03010	6.71154	6.50271	0.01077	0.09995
EEE31154	5.03010	6.71154	6.50271	0.01077	0.09995
EFZ17875	5.03010	6.71154	6.50271	0.01077	0.09995
EGT39833	5.03010	6.71154	6.50271	0.01077	0.09995
XP_001301665	5.03010	6.71154	6.50271	0.01077	0.09995
XP_001438334	5.03010	6.71154	6.50271	0.01077	0.09995
XP_001497503	5.03010	6.71154	6.50271	0.01077	0.09995
XP_001622021	5.03010	6.71154	6.50271	0.01077	0.09995
XP_002032980	5.03010	6.71154	6.50271	0.01077	0.09995
XP_002608279	5.03010	6.71154	6.50271	0.01077	0.09995
XP_002730917	5.03010	6.71154	6.50271	0.01077	0.09995
XP_002734366	5.03010	6.71154	6.50271	0.01077	0.09995
XP_002736788	5.03010	6.71154	6.50271	0.01077	0.09995

Figure S4. Gene Ontology (GO) terms with highest significance levels (Fisher's exact test) in (**A**) upregulated and (**B**) downregulated unigenes.

(**A**)

GO.ID	Term	Annotated	Significant	Expected	classic Fisher
GO:0071695	anatomical structure maturation	20	6	0.690000	0.00004
GO:0007165	signal transduction	1395	75	48.390000	0.00001
GO:0055085	transmembrane transport	337	27	11.690000	0.00003
GO:0006414	translational elongation	145	16	5.030000	0.00004
GO:0006614	SRP-dependent cotranslational protein targeting to membrane	91	12	3.160000	0.00006
GO:0048738	cardiac muscle tissue development	49	8	1.700000	0.00024
GO:0047496	vesicle transport along microtubule	28	6	0.970000	0.00032
GO:0010927	cellular component assembly involved in morphogenesis	182	16	6.310000	0.00052
GO:0006415	translational termination	83	10	2.880000	0.00054
GO:0006413	translational initiation	156	14	5.410000	0.00095
GO:0009207	purine ribonucleoside triphosphate catabolic process	513	31	17.800000	0.00142
GO:0042274	ribosomal small subunit biogenesis	25	5	0.870000	0.00144
GO:0007368	determination of left/right symmetry	81	9	2.810000	0.00182
GO:0043270	positive regulation of ion transport	16	4	0.560000	0.00184
GO:0031032	actomyosin structure organization	83	9	2.880000	0.00216
GO:0000184	nuclear-transcribed mRNA catabolic process, nonsense-mediated decay	100	10	3.470000	0.00229
GO:0034470	ncRNA processing	101	10	3.500000	0.00247
GO:0019083	viral transcription	101	10	3.500000	0.00247
GO:0003002	regionalization	195	15	6.760000	0.00298
GO:0002378	immunoglobulin biosynthetic process	10	3	0.350000	0.00412

(**B**)

GO.ID	Term	Annotated	Significant	Expected	classic Fisher
GO:0006338	chromatin remodeling	78	13	5.83000	0.00470
GO:0006333	chromatin assembly or disassembly	85	14	6.35000	0.00380
GO:0051384	response to glucocorticoid stimulus	42	8	3.14000	0.01120
GO:0032320	positive regulation of Ras GTPase activity	28	6	2.09000	0.01540
GO:0043954	cellular component maintenance	14	4	1.05000	0.01680
GO:0070301	cellular response to hydrogen peroxide	14	4	1.05000	0.01680
GO:0042059	negative regulation of epidermal growth	37	7	2.76000	0.01790
GO:0045862	positive regulation of proteolysis	37	7	2.76000	0.01790
GO:0021915	neural tube development	83	12	6.20000	0.01940
GO:0009161	ribonucleoside monophosphate metabolic process	16	4	1.20000	0.02720
GO:0033333	fin development	16	4	1.20000	0.02720
GO:0032967	positive regulation of collagen biosynthetic process	10	3	0.75000	0.03340
GO:0035329	hippo signaling cascade	10	3	0.75000	0.03340
GO:0050686	negative regulation of mRNA processing	10	3	0.75000	0.03340
GO:0002064	epithelial cell development	17	4	1.27000	0.03350
GO:0032092	positive regulation of protein binding	17	4	1.27000	0.03350
GO:0060271	cilium morphogenesis	92	12	6.87000	0.03990
GO:0070192	chromosome organization involved in meiosis	18	4	1.34000	0.04060
GO:0045732	positive regulation of protein catabolic process	35	6	2.61000	0.04270
GO:0001736	establishment of planar polarity	61	8	4.56000	0.08210
